# Stria medullaris innervation follows the transcriptomic division of the habenula

**DOI:** 10.1038/s41598-022-14328-1

**Published:** 2022-06-16

**Authors:** Iris Juárez-Leal, Estefanía Carretero-Rodríguez, Francisca Almagro-García, Salvador Martínez, Diego Echevarría, Eduardo Puelles

**Affiliations:** Instituto de Neurociencias, Universidad Miguel Hernández de Elche-CSIC, 03550 Sant Joan d’Alacant, Alicante, Spain

**Keywords:** Neuroscience, Neural circuits

## Abstract

The habenula is a complex neuronal population integrated in a pivotal functional position into the vertebrate limbic system. Its main afference is the stria medullaris and its main efference the fasciculus retroflexus. This neuronal complex is composed by two main components, the medial and lateral habenula. Transcriptomic and single cell RNAseq studies have unveiled the morphological complexity of both components. The aim of our work was to analyze the relation between the origin of the axonal fibers and their final distribution in the habenula. We analyzed 754 tracing experiments from Mouse Brain Connectivity Atlas, Allen Brain Map databases, and selected 12 neuronal populations projecting into the habenular territory. Our analysis demonstrated that the projections into the medial habenula discriminate between the different subnuclei and are generally originated in the septal territory. The innervation of the lateral habenula displayed instead a less restricted distribution from preoptic, terminal hypothalamic and peduncular nuclei. Only the lateral oval subnucleus of the lateral habenula presented a specific innervation from the dorsal entopeduncular nucleus. Our results unveiled the necessity of novel sorts of behavioral experiments to dissect the different functions associated with the habenular complex and their correlation with the distinct neuronal populations that generate them.

## Introduction

The habenula (Hb), an important brain region linking the limbic forebrain to the midbrain and rostral hindbrain^1^, is divided into lateral habenula (LHb) and medial habenula (MHb) (Andres et al.^5^). It is located in the most dorsal part of the alar plate of prosomere 2 (Puelles and Rubenstein^12^) and receives projections from the forebrain, via the stria medullaris (sm), and projects to the basal mesencephalon and rostral rhombencephalon through the fasciculus retroflexus (fr; Sutherland^2^).

**Figure 1:**
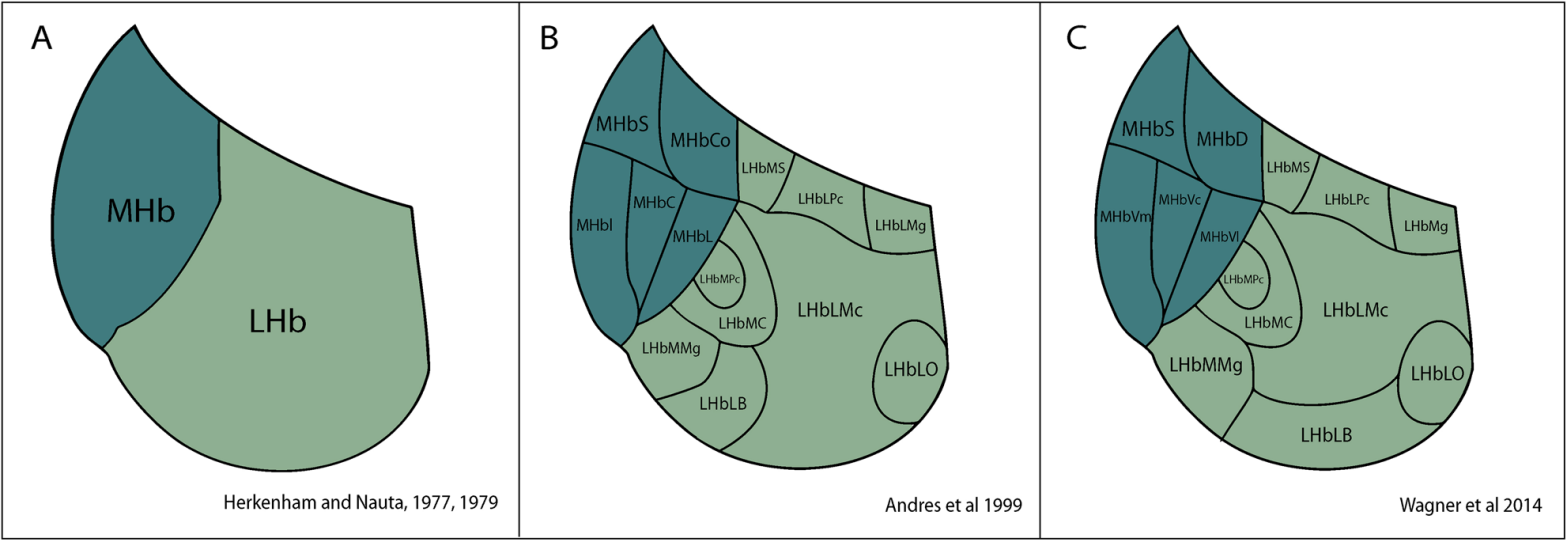
Habenular subdivision scheme. (**A**) Herkenham and Nauta in 1977 and 1979 described the subdivision of the habenular complex in two main components, mHb and lHb. (**B**) Andres and collaborators in 1999 divided the mHb by cytoarchitecture in 5 domains, corresponding the MHbS and the MHbCo to the dorsal mHb and the MHbI, MHbC and the MHbL to the ventral mHb. The lHb was subdivided as well in 9 components that included LHbMS, LHbLPc, LHbLMg in the dorsal aspect, LHbMPc, LHbMC, LHbLMc ald LHbLO in the medial stratum and LHbMMg and MHbLB in the ventral part. (**C**) Wagner and collaborators in 2014 completed this organization by renaming the three ventral components of the mHB as MHbVm, MHbVv and MHbVl and by adding a final subdivision in the ventral aspect of the LHb that was the LHbLB domain. Abbreviations: LHb, lateral habenula; LHbLB: LHbL basal subnucleus; LHbLMc: LHbL magnocellular subnucleus; LHbLO: LHbL oval subnucleus; LHbLPc: LHbL parvocellular subnucleus; LHbMC: LHbM central subnucleus; LHbMPc: LHbM parvocellular subnucleus; LHbMS: LHbM superior subnucleus; MHb: medial habenula; MHbC: MHb central subnucleus; MHbD: MHb dorsal subnucleus; MHbI: Mhb inferior subnucleus; MHbL: MHb lateral subnucleus; MHbS: MHb superior subnucleus; MHbVc: MHb ventral central subnucleus; MHbVl: MHb ventral lateral subnucleus; MHbVm; MHb ventral medial subnucleus.

It was classically divided into medial and lateral portions of the LHb and rostral and caudal portions of the MHb (Herkenham and Nauta^3,4^; Fig. 1A). By cytoarchitectural analysis of the habenular complex in the rat brain, the LHb was later divided into 9 subnuclei and the MHb into 5 subnuclei (Andres et al.^5^; Fig. 1B). This subdivision was confirmed by neurotransmitter distribution^6^. According to topographic, cytochemical, morphological and immunocytochemical criteria, the habenular subdivision described in rat was also shown in the mouse brain^7^. A detailed transcriptomic characterization corroborated the subnuclei previously described in the mouse Hb (Wagner et al.^8^; Fig. 1C). Accordingly, the LHb was described as displaying a medial division that included central (LHbMC), marginal (LHbMMg), parvocellular (LHbMPc) and superior (LHbMS) subnuclei and a lateral division that included: lateral (LHbL), basal (LHbLB), magnocellular (LHbLMc), marginal (LHbLMg), oval (LHbLO) and parvocellular (LHbLPc) subnuclei. The MHb was subdivided into dorsal (MHbD), superior (MHbS), ventral medial (MHbVm), ventral central (MHbVc) and ventral lateral (MHbVl) parts.

In recent years, the development of single cell RNAseq techniques has allowed the study of the expression profile of dissociated habenular neurons^9,10^. These analyses resulted in the identification by a transcriptomical profile of 12 neuronal clusters that largely coincided with the habenular morphological and transcriptomic subdivisions. Some of them belonged to specific subnuclei and others to subdivisions inside a particular subnucleus. This result unveiled the high complexity displayed by the neuronal subpopulations of the Hb complex^9,10^.

The sm is the main afferent tract to the Hb complex. This highly fasciculated tract contains fibers originated from different neuronal populations located in the secondary prosencephalon (hypothalamus and telencephalic vesicle; Herkenham and Nauta^3^; Puelles and Rubenstein^11–13^). The fasciculated sm courses through the most dorsal aspect of the prethalamic prosomere, known as the prethalamic eminence, carrying axons from its multiple origins, caudally into the thalamic prosomere to reach the Hb complex.

It has been described that sm fibers originate in the hypothalamic, pallidal and septal territories. The MHb reportedly receives inputs from the triangular septal nucleus (TRS), the septofimbrial nucleus (SF), the septal area, the bed nucleus of the anterior commissure (BAC), and from hypothalamic entopeduncular neurons^3,14–17^. The LHb, as first described, is innervated by, the entopeduncular nucleus (erroneously identified as basal ganglia and nucleus accumbens), preoptic regions and septum^3^. This description was later confirmed and completed, concluding that the LHb receives inputs from the substantia innominata (SI; Golden et al.^18^; Knowland et al.^19^), dorsal entopeduncular nucleus (EPD)^17,20–22^ and lateral hypothalamic area (LHA)^23–25^. It must be highlighted that it was recently described that almost all the neuronal populations projecting to the Hb in the chick are colonized by tangentially migrated glutamatergic neurons originated from the prethalamic eminence^26,27^. There are three heterochronic migratory streams, by which the prethalamic eminence populates hypothalamic, preoptic, pallidal and septal regions^16,27^. The specific pattern of innervation produced by the different afferent neuronal populations in the LHb or MHb subnuclei has been poorly studied. Only the specific innervation of the LHbLO by the EPD has been described previously^17,28^.

A selective source of innervation thus possibly underlies different functions associated to the Hb components. In general, the MHb has been associated with the mediation of analgesic, autonomic, reward, anxiety and fear responses^29,30^. More specifically, the dorsal aspect of the MHb has been related to exercise motivation, regulation of the hedonic state, intrinsic reinforcement circuit and aversive behaviors^31–33^. The ventral aspect of the MHb has been involved in drug addiction, anxiety, and depression^15,31^. Therefore, the vertebrate MHb is related to emotional behavior^30,32,33^. In contrast, the LHb has been considered as an anti-reward system and appears associated to behavioral and motivational control. In fact, the LHb is involved in regulation of aversion, stress, sleep, mood and maternal behavior^23,34,35^. Results obtained in behavioral experiments point out to a LHb relation with learned helplessness response as well as reward, aversion or punishment behavior^36–40^ and depression^41–45^.

Our present aim is to analyze the possible differential innervation of the multiple habenular subnuclei considering the differential origin of their afferent fibers. The observed distribution was confronted with the subnuclear organization and location of limbic system functions associated to the habenula.

## Results

**Figure 2:**
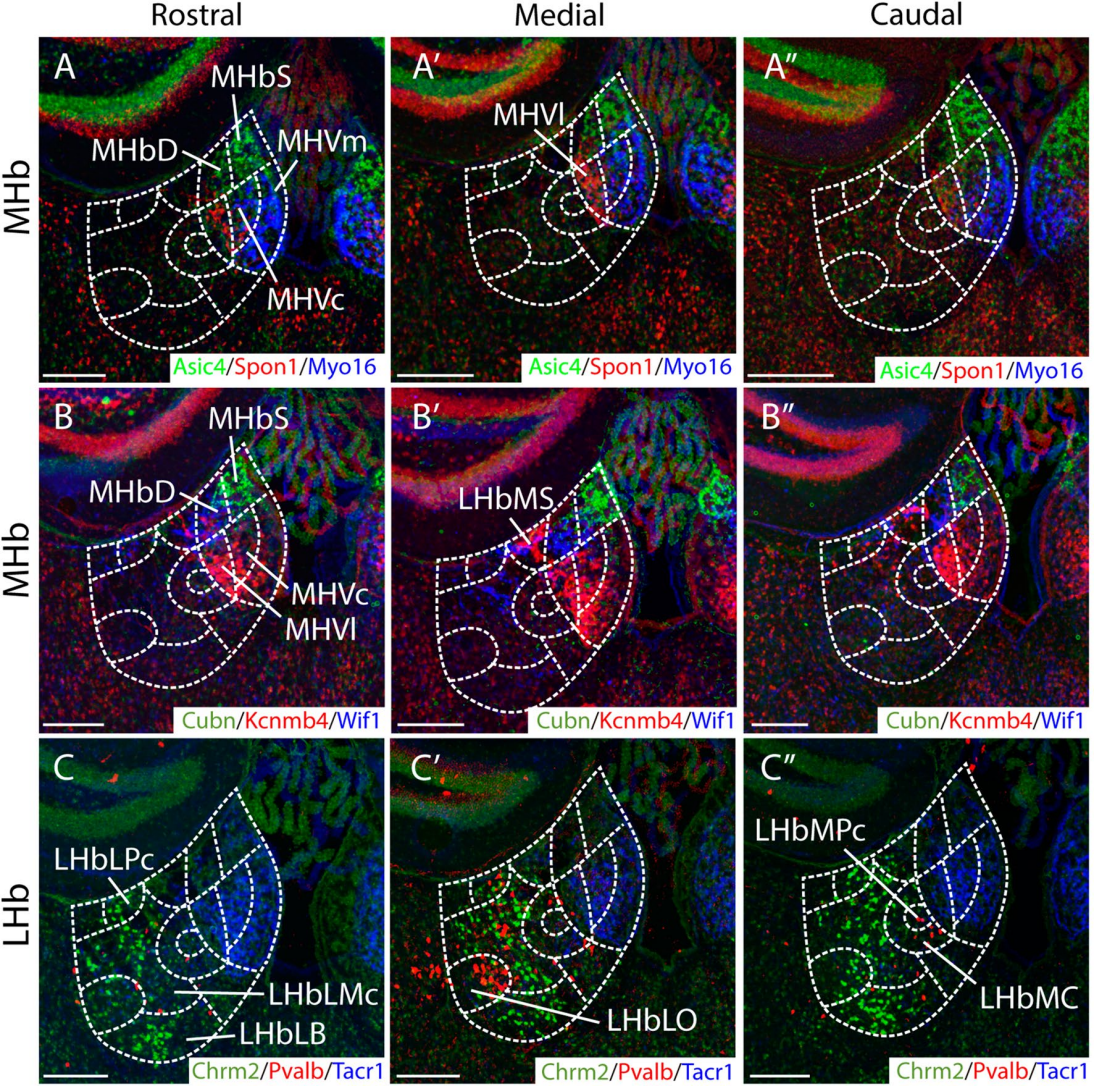
Transcriptomic subdivision of the habenula. (**A**, **A**’, **A**’’) Coronal sections, from rostral to caudal, of adult mouse brain displaying fluorescence overlap of *Asic4*, *Spon1* and *Myo16* gene expression in MHb. *Asic4* is specific of MHbS and MHbD subnuclei and *Spon1* is expressed in MHbVl. While *Myo16* is expressed in MHBVc and MHbVm (**B**, **B**’, **B**’’) Coronal sections, from rostral to caudal, of adult mouse brain displaying fluorescence overlap of *Cubn*, *Kcnmp4* and *Wif1* genes expression. *Cubn* is expressed in the MHbS while *Wif1* is expressed in the MHbD. *Kcnmb4* is mainly expressed in the MHbVl and MHbVc as well as in the LHbMs. (**C**, **C**’ and **C**’’) Coronal sections, from rostral to caudal, of adult mouse brain displaying fluorescence overlap of *Chrm2*, *Pvalb* and *Tacr1* genes. *Chrm2* displayed a scattered pattern in the central LHb, including the LHbLPc, LHbLMc and LHbLB subnuclei. *Pvalb* was expressed in the LHbLO and LHbMPc and LHbMC. *Tacr1* was expressed in the three ventral components of the ventral MHb. Abbreviations: LHb, lateral habenula; LHbLB: LHbL basal subnucleus; LHbLMc: LHbL magnocellular subnucleus; LHbLO: LHbL oval subnucleus; LHbLPc: LHbL parvocellular subnucleus; LHbMC: LHbM central subnucleus; LHbMPc: LHbM parvocellular subnucleus; LHbMS: LHbM superior subnucleus; MHb: medial habenula; MHbD: MHb dorsal subnucleus; MHbS: MHb superior subnucleus; MHbVc: MHb ventral central subnucleus; MHbVl: MHb ventral lateral subnucleus; MHbVm; MHb ventral medial subnucleus. Scale bar: 200 μm. Image credit: Allen Institute for Brain Science. [https://mouse.brain-map.org/].

First, we selected several gene expression patterns, inspired by published single cell RNAseq experiments^9,10^ as examples of Hb subnuclear organization markers (Quina et al.^46^). The images, form rostral to caudal, were color-coded and overlapped by Adobe Software. In the MHb, *Asic4* nicely labelled the MHbS and the MHbD subnuclei, while *Spon1* was expressed in the MHbVl subnucleus and *Myo16* in the remaining MHb ventral components (central and medial; Fig. 2A, A’, A’’). The *Cubn* and *Wif1* expression allowed us to discern between the two dorsal MHb components. Being *Cubn* expressed in the MHbS and *Wif1* in the MHbD (Fig. 2B, B’, B’’). *Kcnmb4* was mainly expressed in the MHbVl and MHbVc allowing us to discern between the positive MHbVc and negative MHbVm (Fig. 2B, B’, B’’), both populations where positive for *Myo16*. *Kcnmb4* was also expressed in the LHbMS (Fig. 2B, B’, B’’). The three ventral components of the MHb shared the expression of *Tacr1* (Fig. 2C, C’, C’’). Finally, in the LHb, *Chrm2* was expressed in the medial region, including the LHbLPc, LHbLMc an LHbLB (Fig. 2C, C’, C’’). While *Pvalb* was expressed in the LHbLO and in the LHbMPc and LHbMC (Fig. 2C, C’, C’’). Once we identified all the different subnuclei of the Hb at the three selected section levels, we proceeded with the analysis of the connectivity experiments.

### Neuronal populations targeting the medial habenula

**Figure 3:**
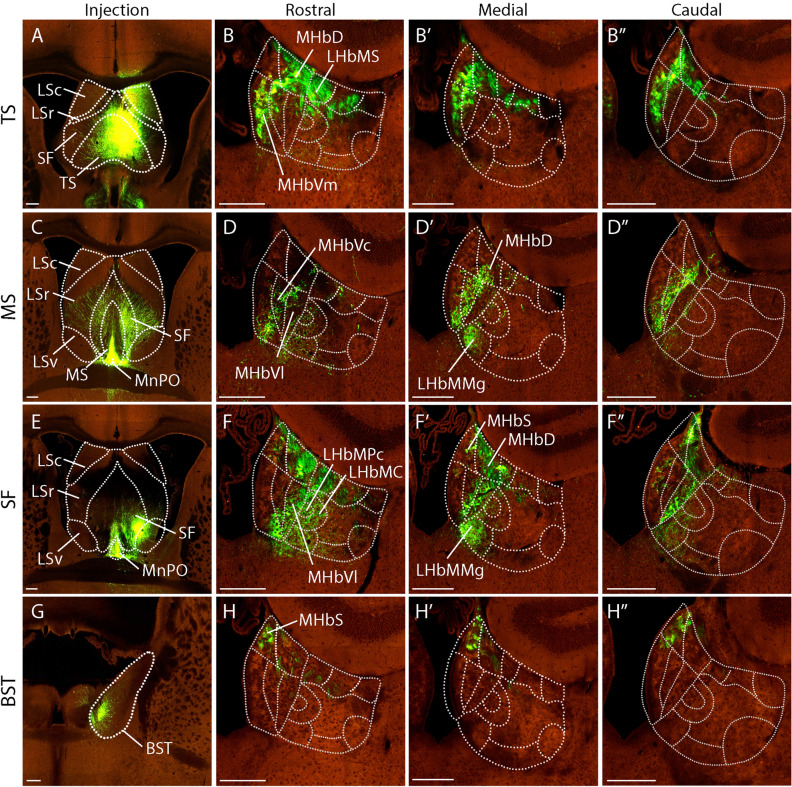
Septal projections to the MHb. (**A**) Injection site in the triangular nucleus of septum (TS; Experiment nº: 125,830,911, identified as Lateral septum rostral part, LSr). (**B**–**B**”) Adult mouse habenula coronal sections, from rostral to caudal, showing the fiber distribution originated from the TS nucleus. (**C**) Injection site in the medial septal nucleus (MS; Experiment nº: 147,162,736). (**D**–**D**”) Adult mouse brain coronal sections of the habenula rostral to caudal, displaying the fibers distribution coming from the MS nucleus. (**E**) Injection site in the septofimbrial nucleus (SF; Experiment nº: 554,021,622). (**F**–**F**”) Adult mouse brain coronal sections of the habenula rostral to caudal, showing the fibers distribution originated from the SF nucleus. (**G**) Injection site in the bed nuclei of the stria terminalis (BST; Experiment: nº 159,433,187. (**H**–**H**”) Adult mouse brain coronal sections of the habenula rostral to caudal, displaying the fibers distribution coming from the BST nucleus, being the only nucleus that is not from septal territory. Abbreviations: BST: bed nuclei of the stria terminalis; LSc: lateral septal nucleus, caudal part; LSr: lateral septal nucleus, rostral part; MS: medial septal nucleus; LHbLMc: LHbL magnocellular subnucleus; LHbMMg: LHbM marginal subnucleus; LHbMPc: LHbM parvocellular subnucleus; MHbD: MHb dorsal subnucleus; MHbS: MHb superior subnucleus; MHbVc: MHb ventral central subnucleus; MHbVl: MHb ventral lateral subnucleus; MHbVm; MHb ventral medial subnucleus; MnPO: median preoptic nucleus; SF: septofimbrial nucleus; TS: triangular nucleus of septum. Scale bar: 200 μm. Image credit: Allen Institute for Brain Science. [https://connectivity.brain-map.org/].

We confirmed four neuronal populations that target the MHb. They mainly belong to the septal region and are the triangular septal nucleus (TS), the medial septal nucleus (MS), the septofimbrial nucleus (SF) and the bed nucleus of the stria terminalis, mediocentral division (BST). The TS (Fig. 3A) projects to the MHbVm, LHbMS and the MHbD. The GFP + axonal terminals extend along the anteroposterior axis of the habenular complex (Fig. 3B–B´´). The proximity of the MS and the median preoptic nucleus (MnPO) did not allow us to find experiments restricted to MS (Fig. 3C). In a first analysis MS/MnPO terminals reached the MHbVc and partially the MHbVm and MHbD (Fig. 3D–D´´). The injections in the SF also affected the MnPO (Fig. 3E). The terminal labelling overlap between the MS and SF experiments allowed us to ascribe the MnPO projection area to the LHbMMg (Fig. 3D´ and F´). The SF nucleus would thus project to the MHbVl and the MHbD (Fig. 3F–F´´). The contralateral MHbS was partially labelled due to positive fibers crossing through the habenular commissure (Fig. 3F´´) SF also projected to the LHb, with terminals found in the LHBMPc and LHbMC (Fig. 3F). Finally, we identified an injection in the BST (Fig. 3G), medio central division, that specifically labelled the MHbS (Fig. 3H–H´´).

In summary, the MHb dorsal area is principally innervated by TS, SF and medio central BST while the three ventral MHb subnuclei have differential innervations. The medial part is innervated by the TS, the central part by the MS and the lateral part by the SF. Note that in the case of the MHbD we cannot exclude that some of the positive fibers are not terminals but passaging fibers to others nuclei due to the location of the sm.

### Neuronal populations targeting the lateral habenula

The LHb receives projections originated from different brain regions. We detected terminals originated from the preoptic area and others parts of the subpallium, terminal and peduncular hypothalamus. The GFP + fibers displayed a diffuse distribution in the LHb when compared to the MHb pattern.

**Figure 4:**
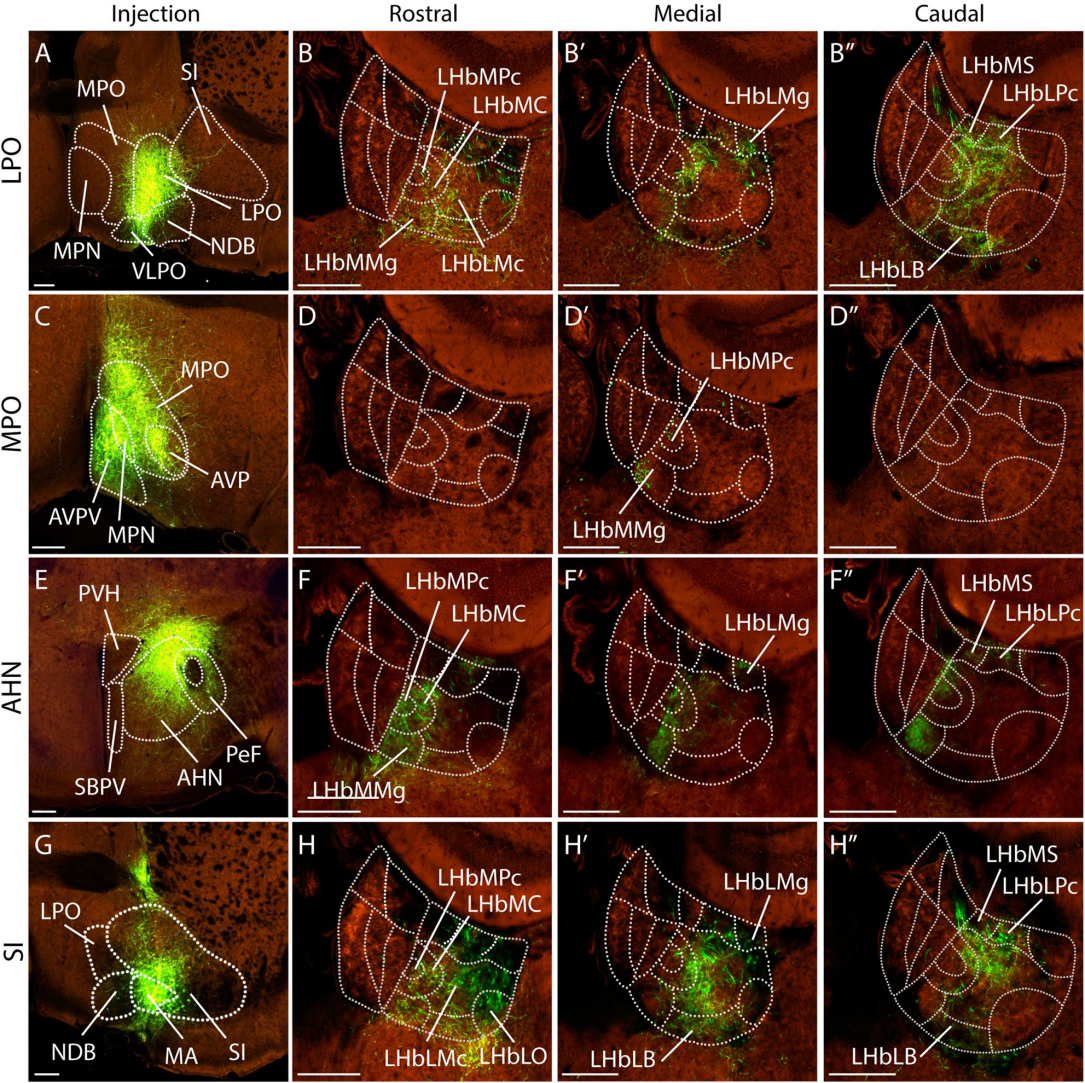
Preoptic area, terminal hypothalamic and pallidal projections to the LHb. (**A**) Injection site in the lateral preoptic area (LPO; Experiment nº: 293,942,188). (**B**–**B**”) Adult mouse habenula coronal sections, from rostral to caudal, showing the fibers distribution originated from the LPO nucleus. (**C**) Injection site in the medial preoptic area (MPO; Experiment nº: 299,247,009). (**D**–**D**”) Adult mouse habenula coronal sections, from rostral to caudal, displaying the fibers distribution coming from the MPO nucleus. (**E**) Injection site in the anterior hypothalamic nucleus (AHN; Experiment nº: 292,035,484). (**F**–**F**”) Adult mouse habenula coronal sections, from rostral to caudal, showing the fibers distribution originated from the AHN nucleus. (**G**) Injection site in the substantia innominata (SI; Experiment nº: 302,739,608). (**H**–**H**”) Adult mouse habenula coronal sections, from rostral to caudal, showing the fiber distribution originated from the SI nucleus. Abbreviations: AHN: anterior hypothalamic nucleus; AVPV: anteroventral periventricular nucleus; AVP: Anteroventral preoptic nucleus; LHb, lateral habenula; LHbLB: LHbL basal subnucleus; LHbLMc: LHbL magnocellular subnucleus; LHbLMg: LHbL marginal subnucleus; LHbLO: LHbL oval subnucleus; LHbLPc: LHbL parvocellular subnucleus; LHbM: LHb medial territory; LHbMC: LHbM central subnucleus; LHbMMg: LHbM marginal subnucleus; LHbMPc: LHbM parvocellular subnucleus; LHbMS: LHbM superior subnucleus; LPO: lateral preoptic area; MA: magnocellular nucleus; MHb: medial habenula; MHbD: MHb dorsal subnucleus; MPO medial preoptic area; MPN: medial preoptic nucleus; NDB: diagonal band nucleus; PeF: perifornical nucleus; SBPV: subparaventricular zone; SI: substantia innominate; VLPO: ventrolateral preoptic nucleus. Scale bar: 200 μm. Image credit: Allen Institute for Brain Science. [https://connectivity.brain-map.org/].

We found four populations from the preoptic area, terminal hypothalamus and subpallial area: the lateral preoptic area (LPO), the medial preoptic area (MPO) and the anterior hypothalamic nucleus (AHN) and the substantia innominate (SI). The LPO injection in the preoptic area (Fig. 4A) illustrated the main projection into the LHbLMc and LHbLB (Fig. 4B, B´´). We also found scattered axons in the LHbMMg, LHbMPc and LHbMC (Fig. 4B–B´´). Some fibers were also detected in the dorsal LHb including LHbMS, LHbLPc and LHbLMg. Therefore, LPO fibers innervate the medial-central and dorsal LHb areas. The MPO injections always included surrounding territories. We selected an injection that affected partially the MPO and also labelled the medial preoptic nucleus (MPN; Fig. 4C). The database contains specific MPN injections that did not display any habenular projections. Therefore, the projections observed only in the medial aspect of the LHb, concerning the LHbMMg and LHbMPc must be due largely to the MPO (Fig. 4D–D´´). In the terminal hypothalamus, the AHN injection (Fig. 4E), that also affected a perifornical nucleus (PeF; peduncular hypothalamic population) displayed projections into the medial area of the LHb, including the LHbMMg, LHbMPc and LHbMC subnuclei (Fig. 4F–F´´). Thus, the LHb medial territory is mainly innervated by LPO, MPO and AHN populations. The LPO also targets the LHb central territory (Fig. 4B–B´´). The subpallial area contains a neuronal population that targets the LHb, namely SI, intermediate stratum of the diagonal domain. The injection in the SI labelled the magnocellular preoptic nucleus (MA; Fig. 4G), and the fibers were distributed in a diffuse pattern throughout the LHb (Fig. 4H–H´´). In the latter´s medial part, the axons concentrated in the LHbLB and LHbLMc subnuclei (Fig. 4H´) and in its caudal part, the fibers also occupied the LHbMS and LHbLPc (Fig. 4H´´).

**Figure 5:**
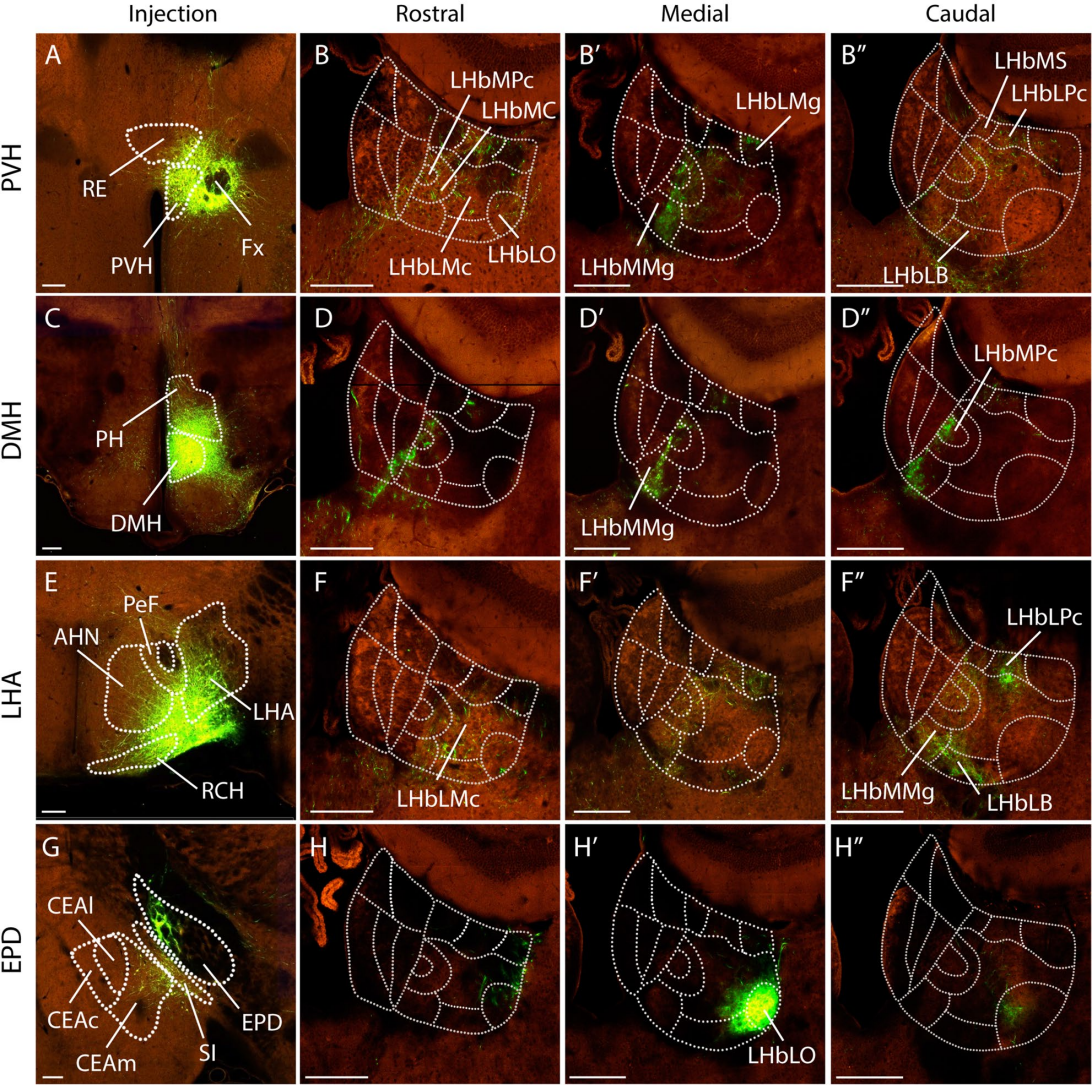
Peduncular hypothalamic projections to the LHb. (**A**) Injection site in the paraventricular hypothalamic nucleus (PVH; Experiment nº: 581,641,279). (**B**–**B**”) Adult mouse habenula coronal sections, from rostral to caudal, showing the fiber distribution originated from the PVH nucleus. (**C**) Injection site in the dorsomedial nucleus of the hypothalamus (DMH; Experiment nº: 178,283,239). (**D**–**D**”) Adult mouse habenula coronal sections, rostral to caudal, displaying the fibers distribution coming from the DMH nucleus. (**E**) Injection site in the lateral hypothalamic area (LHA; Experiment nº: 485,239,207). (**F**–**F**”) Adult mouse habenula coronal sections, rostral to caudal, showing the fibers distribution originated from the LHA nucleus. (**G**) Injection site in the dorsal entopeduncular nucleus (EDP; Experiment nº: 539,498,984, identified as internal segment of globus palidus; GPi). (**H**–**H**”) Adult mouse habenula coronal sections, rostral to caudal, displaying the fibers distribution coming from the EPD nucleus. Abbreviations: AHN: anterior hypothalamic nucleus; DMH: dorsomedial nucleus of the hypothalamus; EPD, dorsal entopeduncular nucleus; Fx: fornix; LHA: lateral hypothalamic area; LHb, lateral habenula; LHbLB: LHbL basal subnucleus; LHbLMc: LHbL magnocellular subnucleus; LHbLMg: LHbL marginal subnucleus; LHbLO: LHbL oval subnucleus; LHbLPc: LHbL parvocellular subnucleus; LHbM: LHb medial territory; LHbMC: LHbM central subnucleus; LHbMMg: LHbM marginal subnucleus; LHbMPc: LHbM parvocellular subnucleus; LHbMS: LHbM superior subnucleus; PH: posterior hypothalamic nucleus; PVH: paraventricular hypothalamic nucleus; RCH: retrochiasmatic area; RE: nucleus of reuniens. Scale bar: 200 μm. Image credit: Allen Institute for Brain Science. [https://connectivity.brain-map.org/].

Four neuronal populations were identified in the peduncular hypothalamus: the paraventricular hypothalamic nucleus (PVH), the dorsomedial hypothalamic nucleus (DMH), the lateral hypothalamic area (LHA) and the dorsal entopeduncular nucleus (EPD). The PVH injection (Fig. 5A) demonstrated a strongly diffuse projection into all the LHb (Fig. 5B–B´´). Only in the LHb medial region, the GFP + fibers displayed a more specific pattern within the LHbMMg, LHbMC and LHbMPc (Fig. 5B´). The DMH injection (Fig. 5C) showed a specific terminal pattern that affected mainly the medial LHb territory, including likewise the LHbMMg, LHbMPc and LHbMC (Fig. 5D–D´´). The LHA was labelled at the level of the AHN and the resulting projection (Fig. 5E) displayed again a diffuse distribution in the LHb territory (Fig. 5F, F´). Caudally, the terminals concentrated in the LHb central territory particularly in LHbMMg, LHbLB and LHbLPc (Fig. 5F–F´´). Accordingly, the DMH targets the LHb medial area while the PVH and LHA distribute in the LHb central territory. The EPD injection (Fig. 5G) labelled fibers that specifically innervated the medial portion of the LHbLO (Fig. 5H´) with a minor projection into neighboring rostral and caudal LHb parts (Fig. 5H–H´´).

### Habenular neuronal cell type distribution

The Hb single cell RNAseq experiments^9,10^ demonstrated the presence of various neurotransmitters-related cell types in this neuronal complex. We checked these neurotransmitter-related patterns testing whether their distribution coincides with habenular subnuclear subdivisions.

**Figure 6:**
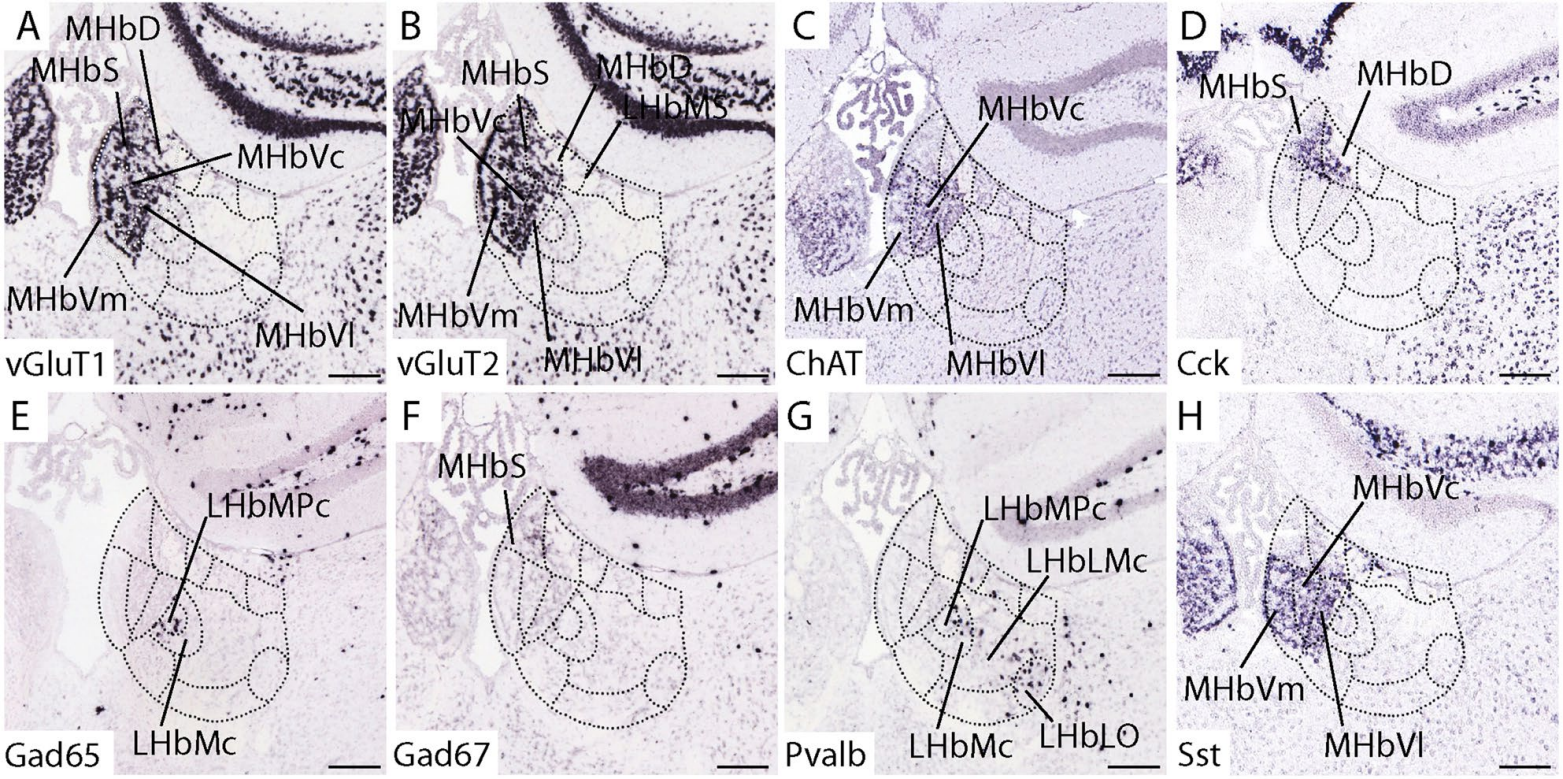
Habenular neuronal cell type distribution. Adult mouse habenula coronal sections displaying the location of specific cell type markers by in situ hybridization. Excitatory neurotransmitters (**A**–**D**). (**A**) *vGlut1* gene, displayed in ventral and dorsal MHb; (**B**) *vGlut2* gene, displayed principally in the MHb and in a the LHb as a scattered pattern. (**C**) *ChAT* gene, displayed in the ventral MHb. (**D**) *Cck* gene, expressed in the dorsal MHb. Inhibitory neurotransmitters (**E**–**H**). (**E**) *Gad65* gene, displayed in LHb with a specific expression in LHbMPc and LHbMc subnuclei. (**D**) *Gad67* gene, displayed in a specific pattern in the MHbS subnulcei. (**E**) *Pvalb* gene, expressed in specific pattern in the LHbMPc, LHbMc and LHbLO subnulcei, also some scattered positive cells were detected in LHbLMc. (**D**) *Sst* gene, displayed in the ventral MHb part. Abbreviations: LHb, lateral habenula; LHbLMc: LHbL magnocellular subnucleus; LHbLO: LHbL oval subnucleus; LHbM: LHb medial territory; LHbMC: LHbM central subnucleus; LHbMPc: LHbM parvocellular subnucleus; LHbMS: LHbM superior subnucleus; MHb: medial habenula; MHbD: MHb dorsal subnucleus; MHbS: MHb superior subnucleus; MHbVc: MHb ventral central subnucleus; MHbVl: MHb ventral lateral subnucleus; MHbVm; MHb ventral medial subnucleus. Scale bar: 200 μm. Image credit: Allen Institute for Brain Science. [https://mouse.brain-map.org/].

Excitatory glutamatergic neurons were predominant in both MHb and LHb. *vGluT1* signal was prevalent in all the MHb (Fig. 6A) while *vGluT2* expression appeared in both habenular nuclei (Fig. 6B). *Choline acetyltransferase* expression labelled cholinergic neurons distributed in the ventral MHb subnuclei (MHbVm, MHbVc and MHbVl; Fig. 6C). *Cholecystokinin* signal was restricted to the dorsal MHb (MHbS and MHbD; Fig. 6D). The inhibitory marker *Gad65* appeared in LHbMPc and partially also in LHbMC as dispersed positive cells (Fig. 6E), while *Gad67* transcripts were localized specifically in the MHbS (Fig. 6F). *Parvalbumin*, specific marker of a subtype of inhibitory gabaergic neurons, was expressed in LHbMPc, LHbMc, LHbLMc and in LHbLO (Fig. 6G). Finally, *Somatostatin* was located in MHbVm, MHbVc and MHbVl as well as in the LHbMS and LHbLPc (Fig. 6H).

Therefore, specialized neurons with specific neurotransmitters are grouped in the different subnuclei described by transcriptomic methodology, and they also are innervated by different neuronal populations.

## Discussion

The single cell transcriptomic RNAseq studies of Hashikawa et al.^10^ and Wallace et al.^9^ have corroborated different subnuclear components of the habenular complex. The MHb is divided into a dorsal part that includes the MHbD and MHbS subnuclei and a ventral part composed by the MHbVm, MHbVc and MHbVl units. On the other hand, the LHb is divided into a dorsal region that involves the LHbMS, LHbLPc and LHbLMg, a central part that includes medial LHbMPc and LHbMC components, a central LHbLMc component and a lateral LHbLO portion, and finally a ventral portion with LHbMMg and LHbLB units. These studies identified the MHb subnuclei by the expression of a specific gene but the LHb components were recognized by a combination of several gene expression patterns. Each distinct MHb component displays internal homogeneity, while the LHb subnuclei show substantial internal heterogeneity. The diverse nature of their neurons indicates an intricated mode of development. It may be hypothesized that the LHb subnuclei are composed of different subsets of neurons that occupy diverse destinations by differential migration processes.

**Figure 7:**
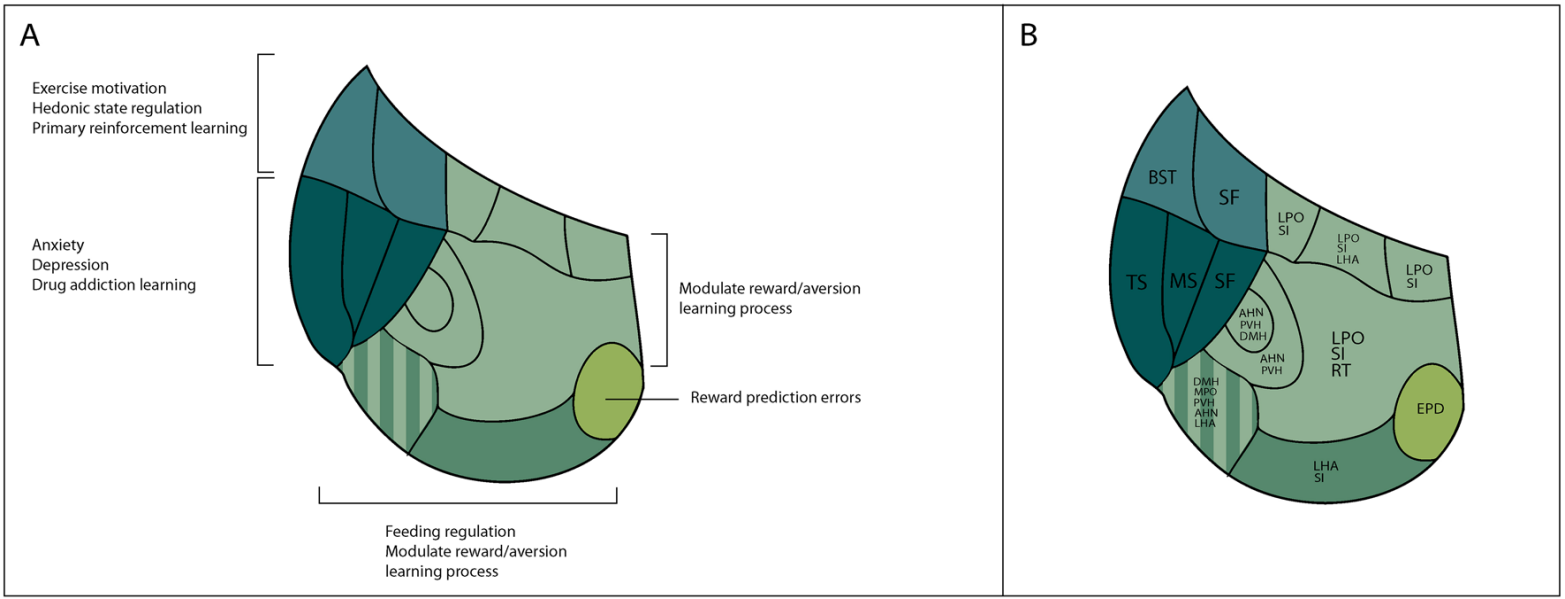
Habenular functions scheme. (**A**) Representation of the habenular functions in relation with the different habenular territories. The limbic functions associated to the habenula are strongly linked with specific subnuclei. (**B**) Representation of the projecting neuronal populations in the different habenular subnuclei. Both schemes allowed us to link projecting populations with the different functions associated to the habenular subnuclei. Abbreviations: AHN: anterior hypothalamic nucleus; BST: bed nuclei of the stria terminalis; DMH: dorsomedial nucleus of the hypothalamus; EPD, dorsal entopeduncular nucleus; LHA: lateral hypothalamic area; LPO: lateral preoptic area; MPO medial preoptic area; MPO medial preoptic area; MS: medial septal nucleus; PVH: paraventricular hypothalamic nucleus; SF: septofimbrial nucleus; SI: substantia innominata; TS: triangular nucleus of septum.

In relation to habenular afferences, the MHb is innervated by four neuronal populations (BST, SF, TS and MS). It is remarkable that almost all the neuronal populations projecting to the MHb belong to the septal territory. This innervation is strongly compartmentalized and the four different sets of axons target specific MHb subnuclei. The BST (medio central division) specifically innervates the MHbS, the SF targets the MHbVl and the MHbD, the TS reaches the MHbD and MHbVm and finally, the MS innervates the MHbVc. It has been described that the TS innervation of the MHb is accompanied by fibers from the bed nucleus of the anterior commissure (BAC; Yamaguchi et al.^47^; Watanabe et al.^16^), but no specific BAC injection was found in the Allen database. Therefore, the dorsal MHb is under the influence of BST, TS and SF, while the ventral MHb is controlled by SF, TS and MS. Functionally, the dorsal MHb region (MHbD and MHbS) is related to exercise motivation, hedonic state regulation and primary reinforcement learning^30,32,33^. Therefore, these functions may be regulated by BST, TS/BAC and SF innervation. The fact that both subnuclei are innervated by different neuronal populations suggests that the functions related to them may be separated between both subnuclei. New and more selective behavioral experiments are needed to dissect the specific function of each dorsal MHb subnucleus. The ventral MHb region is related to anxiety, depression and drug addiction learning^47^. This MHb territory is subdivided in three subnuclei from medial to lateral (MHbVm, Vc and Vl). These subnuclei are each innervated by selective neuronal populations. The ventromedial, central and lateral subnuclei are projected upon by the TS/BAC, MS and SF, respectively. This selective innervation also indicates, as in the dorsal MHb, that each ventral subnucleus may be involved in a different function or that collaboration among them is needed for the cited behavioral phenomena (Fig. 7A, B). As stated before, more specific behavioral experiments must be done to properly understand the role of each ventral MHb subnuclei.

The LHb afferences displayed a wide range of origins. The axonal distributions were less specific for the different LHb subnuclei and usually displayed a diffuse pattern. Nevertheless, we noted that preoptic and AH projections cover the medial aspect of the LHb, whereas diverse afferents from peduncular hypothalamic nuclei reach preferentially the central territory, and SI, EPD and reticular nucleus projections target the lateral components of the LHb. This relatively diffuse pattern of innervation coincides with the complex internal subnuclear organization described by the transcriptomic RNAseq experiments^8–10^. In contrast with the MHb neuronal clusters, the LHb subnuclei required a combination of markers for characterization. We mentioned above the possibility of intermixed neuronal tangential migrations within the LHb components as a plausible explanation of their peculiar partly shared characteristics.

We identified the LPO and MPO in the preoptic area, the AH nucleus in the alar terminal hypothalamus and SI in the subpallial area as territories that project into the LHb. Their axons converged within the medial LHb territory. The LPO projection into the LHb reportedly includes gabaergic and glutamatergic neurons. The balance between these two neurotransmitters modulates the reward/aversion equilibrium in the learning process^48^. The MPO and AH projections into the LHb were previously described^49,50^ but without reference to specific subnuclei or habenular function (Fig. 6A, B). We located PVH, DMH and LHA fibers from the peduncular hypothalamus in the central area of the LHb. The LHA projection into the LHb was previously described^25,51,52^ and was related with feeding regulation and reward/aversion equilibrium in the learning process^25^. No specific information about the PVH and DMH projections into the LHb was found (Fig. 7A,B). Finally, we identified EPD and SI input to the LHb. These nuclei distribute their projections preferentially from medial to lateral areas of the LHb. It is remarkable that the LHbLO is distinguished among all the LHb nuclei due to a strongly specific innervation by the EPD. The LHbLO neurons, together with the LHbMS, display unique electrophysiological properties when compared to the rest of LHb neurons. They respond to Dopamine with an increment of their firing rate in contrast with the rest of LHb neurons^53^. Therefore, this unit constitutes not only a differentiated morphological structure of the LHb but a distinctive functional entity by itself^17,53^. The EPD excitatory projections into LHbLO are related with reward prediction errors modulated by neurotransmitters (Shabel et al.^22^; Wallace et al.^17^; Fig. 7A,B). The high quality of Allen Brain Atlas images allowed a high level of magnification. In most of the experiments analyzed we were able to detect varicosities in the positive fibers that could point to axonal boutons. Nevertheless, in order to confirm this fact, double labeling with synaptic proteins should be perform to confirm the presence of axonal boutons in the different territories.

It must be highlighted that it was recently described in chick that the prethalamic eminence, the dorsal subregion of prethalamus found just rostral to the habenular thalamic region, contributes excitatory neurons by tangential migration during embryonic development to almost all the populations described as projecting into the Hb complex^26,27^. A migratory origin in the prethalamic evidence was demonstrated for the mouse habenulopetal BAC nucleus^16^. The described migration into the peduncular hypothalamic EPD formation nicely explains that this mixed excitatory/inhibitory neuronal population has usually been wrongly assigned to the pallidal territory as part of the rodent GPi^9,17,26,27^. Therefore, the functions assigned to the pallidal GPi^20,22^ seem to correspond to the hypothalamic EPD.

It must be noted that our analysis presents certain limitations due to the fact that we have only used the data obtained from Allen Brain database. In some of the cases it would have been needed more specific and accurate injections or the use of specific viral tracers to label specific neuronal types. Nevertheless, we do not foresee that these specific experiments would strongly modify the conclusions of our work.

The molecular neuronal heterogeneity among the MHb and LHb subnuclei correlates with the distribution of different neurotransmitters. In general, the LHb is divided in two areas (medial and lateral) attending to its different psychobiological functions. The lateral area is related to avoidance behavior to aversive stimuli while the medial part has been involved in despair, helplessness, anhedonia responses and in sleep and circadian rhythms^54^. Our hodological results open the possibility to develop research lines that uncover the specific roles of the different subnuclei of both LHb and MHb.

## Methods

### Allen brain atlas

The Allen Mouse Brain Atlas (© 2021 Allen Institute for Brain Science. Mouse Brain Connectivity. Available at: https://connectivity.brain-map.org) offers Adult Mouse Connectivity Atlas as an image database of axonal projections labeled by viral (rAAV) tracers and visualized using serial two-photon tomography from 2994 experiments.

This resource contains several tools to search through its experiments. The Source Search tool, allows the search of experiments by injection site (Filter source Structures) filtered by mouse line, tracer type and the presence of Intrinsic Signal Images. The Target Search tool allows a "virtual retrograde" search that localizes experiments based on projections located in the structures of interest. Finally, the Spatial Search allows the user to choose either a target signal or injection site based on a voxel selection that retrieves all the experiments with positive signal. The injection summary includes primary and secondary injection structures, the stereotaxic injection Bregma coordinates, the mouse strain, tracer type and the calculated injection summary (%) for the rAAV. The Image Viewer allows to browse the experiment in 2-D and panning through the 140 coronal slices of each experiment. The histogram shows the quantified signal in each structure either by projection volume (mm^3^) or by projection density, which means the fraction of the area occupied by signal compared to the whole structure. The mouse strains used included wild type and transgenic cre lines. Nevertheless, the rAAV used did not include specific sequences to interact with the cre endonuclease.

### Adult mouse connectivity atlas

At the identification stage, 754 experiments (Supplementary Table S1) were revised, using the injection site search, from Septal (30), Hypothalamic (258), Pallidal (67), Striatal (131), and Thalamic (268) areas, according with the habenula-afferent nuclei identified previously. These nuclei were screened by checking both the section images and the projection density window (3D viewer) of each experiment, in order to corroborate the labelled terminations in the habenula. The coronal slices from each confirmed case were reexamined to check the labelled fiber pathway and the terminations in both MHb and LHb.

For the selection of experiments, the following criteria were used: virus volume injected < 0.2 mm^3^ and injection coordinates within anatomical boundaries of the core of interest. Experiments with a massive virus volume injected, or labelling of 4 or more structures to the area of interest, were excluded. In order to systematize the image selection through the Hb between the experiments, we selected three coronal section levels taken 3, 6 and 9 sections rostrally to the habenular commissure. The entire process was carried out through peer analysis, both the selection and screening of the experiments was carried out by two researchers, according to the inclusion and exclusion criteria. For this reason, once all the experiments were collected, each researcher selected one or two experiments from each set that met the criteria. After the first screening, both researchers pooled the results to reach a consensus list of experiments that were suitable in relation to the selection criteria. The experiment number, the amount of virus injected and the Bregma coordinates of the injection were noted (Supplementary Table S1).

All the gene expression images were downloaded from Allen Institute for Brain Science. [https://mouse.brain-map.org/], Mouse Brain (ISH Data). Adobe Photoshop (version 22.1.1) was used for the photo editing program and Adobe Illustrator (version 25.1) was used to generate the figures.

## Supplementary Information

Supplementary Information.

## Data Availability

The datasets generated and/or analyzed during the current study are available in The Allen Mouse Brain Atlas (© 2021 Allen Institute for Brain Science. Mouse Brain Connectivity and Mouse Brain Map (Available at: https://connectivity.brain-map.org and https://mouse.brain-map.org) repository. The accession number to each experiment are contained in Supplementary Table S1.
