# Supplementary material for: Stria medullaris innervation follows the transcriptomic division of the habenula
Source: Sci Rep. 2022 Jun 16;12:10118. doi: 10.1038/s41598-022-14328-1 (PMC9203815; doi:10.1038/s41598-022-14328-1)
Supplement: Supplementary file 1 — Supplementary Information. [file 41598_2022_14328_MOESM1_ESM.pdf]

Supplementary Table S1

Selected and revised connectivity experiments

The first table represent the selected experiments from The Allen Mouse Brain Atlas (© 2021 Allen Institute for Brain Science. Mouse Brain Connectivity. Available at: https://connectivity.brain-map.org). It recollects all the information related to the experiments displayed in the Figures 2-4. The second table represent all the revised experiments, from The Allen Mouse Brain Atlas (© 2021 Allen Institute for Brain Science. Mouse Brain Connectivity, listed by the injected nucleus and grouped by their territory.

Selected experiments

Figure 2: Septal Nuclei (pallial and subpallidal)							
**Nucleus**	**Experiment No.**	**Injection Slice**	**Rostral Slice**	**Medial Slice**	**Caudal Slice**	**Injection Volume**	**Coordinates**
TS	125830911	58/140	67/140	70/140	73/140	0.27 mm^3	Bregma (-0.1, 0.1, 2.3, 0)
SF	147162736	51/140	63/140	66/140	69/140	0.18 mm3	Bregma (0.14, 2.55, 4, 30)
MS	554021622	55/140	68/140	71/140	74/140	0.13 mm3	Bregma (0.62, 0.02, 3.4, 0)
BST	159433187	56/140	65/140	68/140	71/140	0.01 mm3	Bregma (0.1, 0.4, 3.67, 0)

Figure 3: Preoptic area, terminal hypothalamus and basal ganglia							
**Nucleus**	**Experiment No.**	**Injection Slice**	**Rostral Slice**	**Medial Slice**	**Caudal Slice**	**Injection Volume**	**Coordinates**
LPO	293942188	53/140	63/140	66/140	69/140	0.09 mm3	Bregma (0.5, 0.81, 4.84, 0)
MPO	299247009	52/140	65/140	68/140	71/140	0.12 mm3	Bregma (0.5, 0.15, 4.75, 0)
AHN	292035484	65/140	66/140	69/140	72/140	0.19 mm3	Bregma (-0.46, 0.5, 4.8, 0)
SI	302739608	40/140	63/140	66/140	69/140	0.07 mm3	Bregma(0.62, 1.2, 4.85, 0)

Figure 4: Peduncular hypothalamus							
**Nucleus**	**Experiment No.**	**Injection Slice**	**Rostral Slice**	**Medial Slice**	**Caudal Slice**	**Injection Volume**	**Coordinates**
PVH	581641279	59/140	65/140	68/140	71/140	0.07 mm3	Bregma (0.38, 0.02, 4.2, 0)
DMH	178283239	79/140	67/140	70/140	73/140	0.12 mm3	Bregma(-1.5, 0.35, 5.2, 0)
LHA	485239207	65/140	68/140	71/140	74/140	0.10 mm3	Bregma(-0.46, 1.15, 5.45, 0)
EPD	539498984	68/140	67/140	70/140	73/140	0.03 mm3	Bregma(-0.94, -2.25, 4.47, 0)

Experiments revised

Nuclei	Experiment nº	Structure	Region/Part/Area
Gpe	511942270	Pallidum	Dorsal Region
Gpe	265944167	Pallidum	Dorsal Region
Gpe	158373958	Pallidum	Dorsal Region
Gpe	159942097	Pallidum	Dorsal Region
Gpe	647574144	Pallidum	Dorsal Region
Gpe	265943460	Pallidum	Dorsal Region
Gpe	300318924	Pallidum	Dorsal Region
Gpi	305024724	Pallidum	Dorsal Region
Gpi	539498984	Pallidum	Dorsal Region
Gpi	278501857	Pallidum	Dorsal Region
SI	157659671	Pallidum	Ventral Region
SI	161460864	Pallidum	Ventral Region
SI	478491810	Pallidum	Ventral Region
SI	302736304	Pallidum	Ventral Region
SI	126841788	Pallidum	Ventral Region
SI	298835152	Pallidum	Ventral Region
SI	146045723	Pallidum	Ventral Region
SI	159650350	Pallidum	Ventral Region
SI	478581786	Pallidum	Ventral Region
SI	305026861	Pallidum	Ventral Region
SI	147632458	Pallidum	Ventral Region
SI	273026584	Pallidum	Ventral Region
SI	300111087	Pallidum	Ventral Region
SI	126711445	Pallidum	Ventral Region
SI	138058320	Pallidum	Ventral Region
SI	299245589	Pallidum	Ventral Region
SI	549362997	Pallidum	Ventral Region
SI	125436508	Pallidum	Ventral Region
SI	304762245	Pallidum	Ventral Region
MA	No experiments	Pallidum	Ventral Region
NDB	167200755	Pallidum	Medial Region
NDB	286319739	Pallidum	Medial Region
NDB	158257355	Pallidum	Medial Region
NDB	292961470	Pallidum	Medial Region
NDB	515191874	Pallidum	Medial Region
NDB	177605425	Pallidum	Medial Region
NDB	504173156	Pallidum	Medial Region
BST	298835859	Pallidum	Caudal Region
BST	305645843	Pallidum	Caudal Region
BST	308386222	Pallidum	Caudal Region
BST	301947600	Pallidum	Caudal Region
BST	522078446	Pallidum	Caudal Region
BST	241280698	Pallidum	Caudal Region
BST	241279971	Pallidum	Caudal Region
BST	159375036	Pallidum	Caudal Region
BST	176886238	Pallidum	Caudal Region
BST	305026146	Pallidum	Caudal Region

Experiments revised

Nuclei	Experiment nº	Structure	Region/Part/Area
BST	300236763	Pallidum	Caudal Region
BST	265138021	Pallidum	Caudal Region
BST	147049515	Pallidum	Caudal Region
BST	159433187	Pallidum	Caudal Region
BST	204832917	Pallidum	Caudal Region
BST	146747721	Pallidum	Caudal Region
BST	167026321	Pallidum	Caudal Region
BST	117312486	Pallidum	Caudal Region
BST	175739085	Pallidum	Caudal Region
BST	267764292	Pallidum	Caudal Region
BST	304760810	Pallidum	Caudal Region
BST	194948535	Pallidum	Caudal Region
BST	181786681	Pallidum	Caudal Region
BST	171068025	Pallidum	Caudal Region
BST	267763584	Pallidum	Caudal Region
BAC	No experiments	Pallidum	Caudal Region
	**Total**	**Pallidum**	**67**

Nuclei	Experiment nº	Structure	Region/Part/Area
TRS	287950390	Septal	Caudal Part
TRS	182341627	Septal	Caudal Part
SF	147162736	Septal	Caudal Part
SH	No experiments	Septal	Caudal Part
LSc	301672044	Septal	Caudal Part
LSc	527808181	Septal	Caudal Part
LSc	160294327	Septal	Caudal Part
LSc	120436988	Septal	Caudal Part
LSr	267810394	Septal	Rostral Part
LSr	178486024	Septal	Rostral Part
LSr	168094300	Septal	Rostral Part
LSr	300164356	Septal	Rostral Part
LSr	300841699	Septal	Rostral Part
LSr	272917631	Septal	Rostral Part
LSr	293434703	Septal	Rostral Part
LSr	265946352	Septal	Rostral Part
LSr	125830911	Septal	Rostral Part
LSr	301672755	Septal	Rostral Part
LSr	160398593	Septal	Rostral Part
LSr	100141435	Septal	Rostral Part
LSv	113783321	Septal	Rostral Part
LSv	182887258	Septal	Ventral Part
LSv	303537993	Septal	Ventral Part
LSv	167654731	Septal	Ventral Part
MS	265944939	Septal	Ventral Part
MS	113696423	Septal	Ventral Part
MS	156819600	Septal	Ventral Part
MS	278259822	Septal	Ventral Part
MS	100141597	Septal	Ventral Part

Experiments revised

Nuclei	Experiment nº	Structure	Region/Part/Area
MS	303580293	Septal	Ventral Part
MS	554021622	Septal	Ventral Part
	**Total**	**Septal**	**30**
**Nuclei**	**Experiment nº**	**Structure**	**Region/Part/Area**
ACB	168615344	Striatum	Ventral Region
ACB	146470726	Striatum	Ventral Region
ACB	167904966	Striatum	Ventral Region
ACB	171411651	Striatum	Ventral Region
ACB	175373569	Striatum	Ventral Region
ACB	168614604	Striatum	Ventral Region
ACB	300078901	Striatum	Ventral Region
ACB	170785775	Striatum	Ventral Region
ACB	127255254	Striatum	Ventral Region
ACB	175732001	Striatum	Ventral Region
ACB	293008559	Striatum	Ventral Region
ACB	287533790	Striatum	Ventral Region
ACB	265136608	Striatum	Ventral Region
ACB	309740347	Striatum	Ventral Region
ACB	287993060	Striatum	Ventral Region
ACB	300110369	Striatum	Ventral Region
ACB	286486329	Striatum	Ventral Region
FS	No experiments	Striatum	Ventral Region
OT	167904255	Striatum	Ventral Region
OT	292962283	Striatum	Ventral Region
CP	113036264	Striatum	Dorsal Region
CP	513775257	Striatum	Dorsal Region
CP	485846989	Striatum	Dorsal Region
CP	180982124	Striatum	Dorsal Region
CP	117317884	Striatum	Dorsal Region
CP	120762196	Striatum	Dorsal Region
CP	267762146	Striatum	Dorsal Region
CP	159552290	Striatum	Dorsal Region
CP	114399934	Striatum	Dorsal Region
CP	113505468	Striatum	Dorsal Region
CP	272824561	Striatum	Dorsal Region
CP	113766038	Striatum	Dorsal Region
CP	127711803	Striatum	Dorsal Region
CP	160537796	Striatum	Dorsal Region
CP	158916311	Striatum	Dorsal Region
CP	264095536	Striatum	Dorsal Region
CP	160537018	Striatum	Dorsal Region
CP	120570964	Striatum	Dorsal Region
CP	146553266	Striatum	Dorsal Region
CP	127762867	Striatum	Dorsal Region
CP	148198052	Striatum	Dorsal Region
CP	124059700	Striatum	Dorsal Region
CP	159941339	Striatum	Dorsal Region

Experiments revised

Nuclei	Experiment nº	Structure	Region/Part/Area
CP	505807398	Striatum	Dorsal Region
CP	155736539	Striatum	Dorsal Region
CP	159329308	Striatum	Dorsal Region
CP	112307754	Striatum	Dorsal Region
CP	100142580	Striatum	Dorsal Region
CP	265929968	Striatum	Dorsal Region
CP	112458831	Striatum	Dorsal Region
CP	159223001	Striatum	Dorsal Region
CP	127140981	Striatum	Dorsal Region
CP	293366741	Striatum	Dorsal Region
CP	158019342	Striatum	Dorsal Region
CP	126853068	Striatum	Dorsal Region
CP	303478748	Striatum	Dorsal Region
CP	293366035	Striatum	Dorsal Region
CP	183009881	Striatum	Dorsal Region
CP	158020947	Striatum	Dorsal Region
CP	156670520	Striatum	Dorsal Region
CP	307910595	Striatum	Dorsal Region
CP	292620968	Striatum	Dorsal Region
CP	575683020	Striatum	Dorsal Region
CP	175732996	Striatum	Dorsal Region
CP	127224133	Striatum	Dorsal Region
CP	160540013	Striatum	Dorsal Region
CP	287994474	Striatum	Dorsal Region
CP	175072215	Striatum	Dorsal Region
CP	157911832	Striatum	Dorsal Region
CP	301180385	Striatum	Dorsal Region
CP	155737254	Striatum	Dorsal Region
CP	301620241	Striatum	Dorsal Region
CP	514505957	Striatum	Dorsal Region
CP	307909888	Striatum	Dorsal Region
CP	309739641	Striatum	Dorsal Region
CP	161176690	Striatum	Dorsal Region
CP	286649703	Striatum	Dorsal Region
CP	278434443	Striatum	Dorsal Region
CP	287995180	Striatum	Dorsal Region
CP	272697238	Striatum	Dorsal Region
CP	293473098	Striatum	Dorsal Region
CP	286311648	Striatum	Dorsal Region
CP	307692311	Striatum	Dorsal Region
CP	292959343	Striatum	Dorsal Region
CP	310175667	Striatum	Dorsal Region
CP	477924853	Striatum	Dorsal Region
CP	308395312	Striatum	Dorsal Region
AAA	175372863	Striatum	Amygdalar
BA	No experiments	Striatum	Amygdalar
IA	No experiments	Striatum	Amygdalar

Experiments revised

Nuclei	Experiment nº	Structure	Region/Part/Area
CEA	265648940	Striatum	Amygdalar
CEA	127761449	Striatum	Amygdalar
CEA	181890477	Striatum	Amygdalar
CEA	554022330	Striatum	Amygdalar
CEA	304970618	Striatum	Amygdalar
CEA	146795148	Striatum	Amygdalar
CEA	286774064	Striatum	Amygdalar
CEA	278179088	Striatum	Amygdalar
CEA	112459547	Striatum	Amygdalar
CEA	241279261	Striatum	Amygdalar
CEA	573639461	Striatum	Amygdalar
CEA	181889764	Striatum	Amygdalar
CEA	120281646	Striatum	Amygdalar
CEA	539641136	Striatum	Amygdalar
CEA	265945645	Striatum	Amygdalar
CEA	267152406	Striatum	Amygdalar
CEA	277856332	Striatum	Amygdalar
CEA	204907355	Striatum	Amygdalar
CEA	513498584	Striatum	Amygdalar
CEA	543875354	Striatum	Amygdalar
MEA	175072921	Striatum	Amygdalar
MEA	165034344	Striatum	Amygdalar
MEA	180981417	Striatum	Amygdalar
MEA	182041643	Striatum	Amygdalar
MEA	267547788	Striatum	Amygdalar
MEA	170860801	Striatum	Amygdalar
MEA	125361005	Striatum	Amygdalar
MEA	272819994	Striatum	Amygdalar
MEA	168363874	Striatum	Amygdalar
MEA	293469501	Striatum	Amygdalar
MEA	305645132	Striatum	Amygdalar
MEA	157550122	Striatum	Amygdalar
MEA	146985623	Striatum	Amygdalar
MEA	549361039	Striatum	Amygdalar
MEA	304694870	Striatum	Amygdalar
MEA	305124396	Striatum	Amygdalar
MEA	170946889	Striatum	Amygdalar
MEA	286303000	Striatum	Amygdalar
MEA	564357489	Striatum	Amygdalar
MEA	299896150	Striatum	Amygdalar
MEA	309386361	Striatum	Amygdalar
MEA	287666431	Striatum	Amygdalar
MEA	549805072	Striatum	Amygdalar
MEA	303578324	Striatum	Amygdalar
	**Total**	**Striatum**	**131**
**Nuclei**	**Experiment nº**	**Structure**	**Region/Part/Area**
SO	147790922	Hypothalamus	Periventricular Zone

Experiments revised

Nuclei	Experiment nº	Structure	Region/Part/Area
SO	587294457	Hypothalamus	Periventricular Zone
SO	286726065	Hypothalamus	Periventricular Zone
SO	178488152	Hypothalamus	Periventricular Zone
SO	147968866	Hypothalamus	Periventricular Zone
ASO	No experiments	Hypothalamus	Periventricular Zone
PVH	176432524	Hypothalamus	Periventricular Zone
PVH	581641279	Hypothalamus	Periventricular Zone
PVH	127470976	Hypothalamus	Periventricular Zone
PVH	302221478	Hypothalamus	Periventricular Zone
PVH	266840498	Hypothalamus	Periventricular Zone
PVH	183459175	Hypothalamus	Periventricular Zone
PVH	540685246	Hypothalamus	Periventricular Zone
PVH	147136518	Hypothalamus	Periventricular Zone
PVH	112951097	Hypothalamus	Periventricular Zone
PVH	287044088	Hypothalamus	Periventricular Zone
PVH	146983504	Hypothalamus	Periventricular Zone
PVH	267997620	Hypothalamus	Periventricular Zone
Pva	No experiments	Hypothalamus	Periventricular Zone
Pvi	No experiments	Hypothalamus	Periventricular Zone
ARH	263369222	Hypothalamus	Periventricular Zone
ARH	175738378	Hypothalamus	Periventricular Zone
ARH	181891892	Hypothalamus	Periventricular Zone
ARH	176431817	Hypothalamus	Periventricular Zone
ARH	232311236	Hypothalamus	Periventricular Zone
ARH	286726777	Hypothalamus	Periventricular Zone
ARH	178282527	Hypothalamus	Periventricular Zone
ARH	286318327	Hypothalamus	Periventricular Zone
ARH	232310521	Hypothalamus	Periventricular Zone
ARH	146554676	Hypothalamus	Periventricular Zone
ARH	159751184	Hypothalamus	Periventricular Zone
ARH	298105299	Hypothalamus	Periventricular Zone
ARH	171482142	Hypothalamus	Periventricular Zone
ARH	158142090	Hypothalamus	Periventricular Zone
ARH	241278553	Hypothalamus	Periventricular Zone
ARH	146660999	Hypothalamus	Periventricular Zone
ARH	586447435	Hypothalamus	Periventricular Zone
ADP	309794438	Hypothalamus	Periventricular Region
ADP	293114113	Hypothalamus	Periventricular Region
AVP	No experiments	Hypothalamus	Periventricular Region
AVPV	138059031	Hypothalamus	Periventricular Region
DMH	266174751	Hypothalamus	Periventricular Region
DMH	304617742	Hypothalamus	Periventricular Region
DMH	518015408	Hypothalamus	Periventricular Region
DMH	287538943	Hypothalamus	Periventricular Region
DMH	178283239	Hypothalamus	Periventricular Region
DMH	306271212	Hypothalamus	Periventricular Region
DMH	113314337	Hypothalamus	Periventricular Region

Experiments revised

Nuclei	Experiment nº	Structure	Region/Part/Area
DMH	160296448	Hypothalamus	Periventricular Region
DMH	265813096	Hypothalamus	Periventricular Region
DMH	160317628	Hypothalamus	Periventricular Region
DMH	298833739	Hypothalamus	Periventricular Region
DMH	182336846	Hypothalamus	Periventricular Region
MEPO	No experiments	Hypothalamus	Periventricular Region
MPO	158738180	Hypothalamus	Periventricular Region
MPO	113554719	Hypothalamus	Periventricular Region
MPO	175263771	Hypothalamus	Periventricular Region
MPO	119846838	Hypothalamus	Periventricular Region
MPO	182459635	Hypothalamus	Periventricular Region
MPO	298049545	Hypothalamus	Periventricular Region
MPO	294005186	Hypothalamus	Periventricular Region
MPO	158315810	Hypothalamus	Periventricular Region
MPO	299247009	Hypothalamus	Periventricular Region
MPO	277800288	Hypothalamus	Periventricular Region
MPO	292123352	Hypothalamus	Periventricular Region
OV	No experiments	Hypothalamus	Periventricular Region
PD	No experiments	Hypothalamus	Periventricular Region
PS	No experiments	Hypothalamus	Periventricular Region
PVp	168005102	Hypothalamus	Periventricular Region
PVp	264078267	Hypothalamus	Periventricular Region
PVp	168362462	Hypothalamus	Periventricular Region
PVp	167117360	Hypothalamus	Periventricular Region
Pvpo	No experiments	Hypothalamus	Periventricular Region
SBPV	304473503	Hypothalamus	Periventricular Region
SCH	287665706	Hypothalamus	Periventricular Region
SCH	293431163	Hypothalamus	Periventricular Region
VMPO	No experiments	Hypothalamus	Periventricular Region
VLPO	No experiments	Hypothalamus	Periventricular Region
AHN	181057754	Hypothalamus	Medial Zone
AHN	180674463	Hypothalamus	Medial Zone
AHN	523705737	Hypothalamus	Medial Zone
AHN	126116142	Hypothalamus	Medial Zone
AHN	175106053	Hypothalamus	Medial Zone
AHN	182842391	Hypothalamus	Medial Zone
AHN	146660293	Hypothalamus	Medial Zone
AHN	267928135	Hypothalamus	Medial Zone
AHN	159375743	Hypothalamus	Medial Zone
AHN	127649713	Hypothalamus	Medial Zone
AHN	266490034	Hypothalamus	Medial Zone
AHN	286727483	Hypothalamus	Medial Zone
AHN	305092904	Hypothalamus	Medial Zone
AHN	292035484	Hypothalamus	Medial Zone
AHN	301673462	Hypothalamus	Medial Zone
AHN	305677409	Hypothalamus	Medial Zone
AHN	299408890	Hypothalamus	Medial Zone

Experiments revised

Nuclei	Experiment nº	Structure	Region/Part/Area
AHN	300167479	Hypothalamus	Medial Zone
AHN	515198413	Hypothalamus	Medial Zone
LM	298104533	Hypothalamus	Medial Zone
LM	157952778	Hypothalamus	Medial Zone
LM	520619072	Hypothalamus	Medial Zone
MM	158314987	Hypothalamus	Medial Zone
MM	126710740	Hypothalamus	Medial Zone
MM	182182936	Hypothalamus	Medial Zone
MM	307655867	Hypothalamus	Medial Zone
MM	168364580	Hypothalamus	Medial Zone
MM	304720034	Hypothalamus	Medial Zone
MM	287173396	Hypothalamus	Medial Zone
MM	127396760	Hypothalamus	Medial Zone
MM	558673113	Hypothalamus	Medial Zone
MM	304720741	Hypothalamus	Medial Zone
MM	148197327	Hypothalamus	Medial Zone
MM	273055501	Hypothalamus	Medial Zone
SUM	581350498	Hypothalamus	Medial Zone
SUM	114045733	Hypothalamus	Medial Zone
SUM	519164644	Hypothalamus	Medial Zone
SUM	304947804	Hypothalamus	Medial Zone
TMv	520336173	Hypothalamus	Medial Zone
MPN	127909584	Hypothalamus	Medial Zone
MPN	287246555	Hypothalamus	Medial Zone
MPN	120280191	Hypothalamus	Medial Zone
MPN	114472860	Hypothalamus	Medial Zone
MPN	305270515	Hypothalamus	Medial Zone
MPN	294355509	Hypothalamus	Medial Zone
MPN	293549729	Hypothalamus	Medial Zone
MPN	301061596	Hypothalamus	Medial Zone
MPN	301989585	Hypothalamus	Medial Zone
MPN	160399309	Hypothalamus	Medial Zone
MPN	182460343	Hypothalamus	Medial Zone
MPN	587060515	Hypothalamus	Medial Zone
PMd	No experiments	Hypothalamus	Medial Zone
PMv	294316542	Hypothalamus	Medial Zone
PMv	146659588	Hypothalamus	Medial Zone
PMv	299654968	Hypothalamus	Medial Zone
PMv	263780018	Hypothalamus	Medial Zone
PMv	179904203	Hypothalamus	Medial Zone
PMv	520342605	Hypothalamus	Medial Zone
PMv	157952068	Hypothalamus	Medial Zone
PMv	286728896	Hypothalamus	Medial Zone
PMv	515520455	Hypothalamus	Medial Zone
PMv	167656152	Hypothalamus	Medial Zone
PMv	267398651	Hypothalamus	Medial Zone
PMv	298178912	Hypothalamus	Medial Zone

Experiments revised

Nuclei	Experiment nº	Structure	Region/Part/Area
PVHd	299759881	Hypothalamus	Medial Zone
PVHd	304998039	Hypothalamus	Medial Zone
PVHd	300923916	Hypothalamus	Medial Zone
PVHd	166532512	Hypothalamus	Medial Zone
PVHd	147051682	Hypothalamus	Medial Zone
VMH	158258062	Hypothalamus	Medial Zone
VMH	313325371	Hypothalamus	Medial Zone
VMH	267540168	Hypothalamus	Medial Zone
VMH	264319363	Hypothalamus	Medial Zone
VMH	478095541	Hypothalamus	Medial Zone
VMH	176886958	Hypothalamus	Medial Zone
VMH	175106769	Hypothalamus	Medial Zone
VMH	292211026	Hypothalamus	Medial Zone
VMH	573330828	Hypothalamus	Medial Zone
VMH	303708513	Hypothalamus	Medial Zone
VMH	503324388	Hypothalamus	Medial Zone
VMH	277854208	Hypothalamus	Medial Zone
VMH	308641549	Hypothalamus	Medial Zone
VMH	552759734	Hypothalamus	Medial Zone
VMH	540139629	Hypothalamus	Medial Zone
VMH	277854916	Hypothalamus	Medial Zone
VMH	266489212	Hypothalamus	Medial Zone
VMH	503036583	Hypothalamus	Medial Zone
VMH	182337561	Hypothalamus	Medial Zone
VMH	286556208	Hypothalamus	Medial Zone
VMH	114290225	Hypothalamus	Medial Zone
VMH	277615922	Hypothalamus	Medial Zone
VMH	157549402	Hypothalamus	Medial Zone
VMH	286319033	Hypothalamus	Medial Zone
PH	175374275	Hypothalamus	Medial Zone
PH	164986046	Hypothalamus	Medial Zone
PH	573624241	Hypothalamus	Medial Zone
PH	268208632	Hypothalamus	Medial Zone
PH	176898557	Hypothalamus	Medial Zone
PH	159649643	Hypothalamus	Medial Zone
PH	268204599	Hypothalamus	Medial Zone
PH	127710392	Hypothalamus	Medial Zone
PH	551738231	Hypothalamus	Medial Zone
PH	159222295	Hypothalamus	Medial Zone
PH	112425523	Hypothalamus	Medial Zone
PH	278260569	Hypothalamus	Medial Zone
PH	100141434	Hypothalamus	Medial Zone
PH	292480129	Hypothalamus	Medial Zone
PH	293255030	Hypothalamus	Medial Zone
PH	266248065	Hypothalamus	Medial Zone
PH	557973149	Hypothalamus	Medial Zone
PH	267213793	Hypothalamus	Medial Zone

Experiments revised

Nuclei	Experiment nº	Structure	Region/Part/Area
PH	555011865	Hypothalamus	Medial Zone
PH	168616827	Hypothalamus	Medial Zone
PH	302086846	Hypothalamus	Medial Zone
PH	266837456	Hypothalamus	Medial Zone
PH	302087552	Hypothalamus	Medial Zone
PH	300927483	Hypothalamus	Medial Zone
LHA	165035106	Hypothalamus	Lateral Zone
LHA	114046440	Hypothalamus	Lateral Zone
LHA	305379705	Hypothalamus	Lateral Zone
LHA	158373181	Hypothalamus	Lateral Zone
LHA	176887774	Hypothalamus	Lateral Zone
LHA	156195758	Hypothalamus	Lateral Zone
LHA	170860092	Hypothalamus	Lateral Zone
LHA	113313632	Hypothalamus	Lateral Zone
LHA	127470271	Hypothalamus	Lateral Zone
LHA	113444277	Hypothalamus	Lateral Zone
LHA	304674547	Hypothalamus	Lateral Zone
LHA	278508779	Hypothalamus	Lateral Zone
LHA	298048079	Hypothalamus	Lateral Zone
LHA	117302771	Hypothalamus	Lateral Zone
LHA	112372418	Hypothalamus	Lateral Zone
LHA	293368154	Hypothalamus	Lateral Zone
LHA	113369603	Hypothalamus	Lateral Zone
LHA	278510197	Hypothalamus	Lateral Zone
LHA	485239207	Hypothalamus	Lateral Zone
LHA	298048787	Hypothalamus	Lateral Zone
LHA	298078515	Hypothalamus	Lateral Zone
LHA	113506174	Hypothalamus	Lateral Zone
LHA	287667137	Hypothalamus	Lateral Zone
LHA	113225519	Hypothalamus	Lateral Zone
LHA	286882342	Hypothalamus	Lateral Zone
LHA	267213087	Hypothalamus	Lateral Zone
LHA	171020416	Hypothalamus	Lateral Zone
LHA	515410820	Hypothalamus	Lateral Zone
LHA	568768472	Hypothalamus	Lateral Zone
LHA	142653395	Hypothalamus	Lateral Zone
LHA	293254286	Hypothalamus	Lateral Zone
LHA	265125894	Hypothalamus	Lateral Zone
LHA	267396430	Hypothalamus	Lateral Zone
LPO	113553300	Hypothalamus	Lateral Zone
LPO	517975511	Hypothalamus	Lateral Zone
LPO	302016815	Hypothalamus	Lateral Zone
LPO	293942188	Hypothalamus	Lateral Zone
PSTN	165974379	Hypothalamus	Lateral Zone
PSTN	264696942	Hypothalamus	Lateral Zone
PeF	No experiments	Hypothalamus	Lateral Zone
RCH	No experiments	Hypothalamus	Lateral Zone

Experiments revised

Nuclei	Experiment nº	Structure	Region/Part/Area
STN	564688610	Hypothalamus	Lateral Zone
STN	544455391	Hypothalamus	Lateral Zone
STN	146986331	Hypothalamus	Lateral Zone
TU	176888661	Hypothalamus	Lateral Zone
TU	264320076	Hypothalamus	Lateral Zone
TU	264248605	Hypothalamus	Lateral Zone
TU	112228391	Hypothalamus	Lateral Zone
TU	177890956	Hypothalamus	Lateral Zone
TU	547202505	Hypothalamus	Lateral Zone
TU	304948510	Hypothalamus	Lateral Zone
TU	551756337	Hypothalamus	Lateral Zone
TU	267397226	Hypothalamus	Lateral Zone
TU	113037759	Hypothalamus	Lateral Zone
ZI	175018829	Hypothalamus	Lateral Zone
ZI	126190743	Hypothalamus	Lateral Zone
ZI	113095845	Hypothalamus	Lateral Zone
ZI	171066613	Hypothalamus	Lateral Zone
ZI	264697714	Hypothalamus	Lateral Zone
ZI	168663472	Hypothalamus	Lateral Zone
ZI	174788109	Hypothalamus	Lateral Zone
ZI	171065906	Hypothalamus	Lateral Zone
ZI	170263370	Hypothalamus	Lateral Zone
ZI	162018879	Hypothalamus	Lateral Zone
ZI	304858700	Hypothalamus	Lateral Zone
ZI	301539438	Hypothalamus	Lateral Zone
ZI	170858675	Hypothalamus	Lateral Zone
ZI	508539001	Hypothalamus	Lateral Zone
ZI	287492899	Hypothalamus	Lateral Zone
ZI	156315468	Hypothalamus	Lateral Zone
ZI	522255595	Hypothalamus	Lateral Zone
ZI	306270474	Hypothalamus	Lateral Zone
ZI	286662551	Hypothalamus	Lateral Zone
ZI	511549277	Hypothalamus	Lateral Zone
ZI	299760587	Hypothalamus	Lateral Zone
ZI	305321883	Hypothalamus	Lateral Zone
	**Total**	**Hypothalamus**	**258**

Nuclei	Experiment nº	Structure	Region/Part/Area
VAL	113884251	Thalamus	Sensory-motor cortex related
VAL	300237470	Thalamus	Sensory-motor cortex related
VAL	310193233	Thalamus	Sensory-motor cortex related
VM	157063074	Thalamus	Sensory-motor cortex related
VM	174736554	Thalamus	Sensory-motor cortex related
VM	127797441	Thalamus	Sensory-motor cortex related
VM	538834292	Thalamus	Sensory-motor cortex related
VM	267929554	Thalamus	Sensory-motor cortex related
VM	170859382	Thalamus	Sensory-motor cortex related
VM	273025872	Thalamus	Sensory-motor cortex related

Experiments revised

Nuclei	Experiment nº	Structure	Region/Part/Area
VM	306444486	Thalamus	Sensory-motor cortex related
VM	268205344	Thalamus	Sensory-motor cortex related
VM	180707817	Thalamus	Sensory-motor cortex related
VPM	268399868	Thalamus	Sensory-motor cortex related
VPM	478581080	Thalamus	Sensory-motor cortex related
VPM	268206050	Thalamus	Sensory-motor cortex related
VPM	312240825	Thalamus	Sensory-motor cortex related
VPM	277958616	Thalamus	Sensory-motor cortex related
VPM	100141223	Thalamus	Sensory-motor cortex related
VPM	552280478	Thalamus	Sensory-motor cortex related
VPM	158375425	Thalamus	Sensory-motor cortex related
VPM	156202979	Thalamus	Sensory-motor cortex related
VPM	180628971	Thalamus	Sensory-motor cortex related
VPMpc	162018169	Thalamus	Sensory-motor cortex related
VPMpc	301735080	Thalamus	Sensory-motor cortex related
SPFm	278261300	Thalamus	Sensory-motor cortex related
SPFm	159258618	Thalamus	Sensory-motor cortex related
MG	305269070	Thalamus	Sensory-motor cortex related
MG	180520257	Thalamus	Sensory-motor cortex related
MG	178489574	Thalamus	Sensory-motor cortex related
MG	299996344	Thalamus	Sensory-motor cortex related
MG	183329991	Thalamus	Sensory-motor cortex related
LGd	298004028	Thalamus	Sensory-motor cortex related
LGd	292320572	Thalamus	Sensory-motor cortex related
LGd	480692170	Thalamus	Sensory-motor cortex related
LGd	298003295	Thalamus	Sensory-motor cortex related
LGd	479268685	Thalamus	Sensory-motor cortex related
LGd	478258719	Thalamus	Sensory-motor cortex related
LGd	479670988	Thalamus	Sensory-motor cortex related
LGd	100141598	Thalamus	Sensory-motor cortex related
LGd	263241470	Thalamus	Sensory-motor cortex related
LGd	479891303	Thalamus	Sensory-motor cortex related
LGd	278070717	Thalamus	Sensory-motor cortex related
LGd	156198187	Thalamus	Sensory-motor cortex related
LGd	479671695	Thalamus	Sensory-motor cortex related
LGd	642811309	Thalamus	Sensory-motor cortex related
LGd	642177206	Thalamus	Sensory-motor cortex related
LGd	293942897	Thalamus	Sensory-motor cortex related
LGd	266248776	Thalamus	Sensory-motor cortex related
LGd	293914766	Thalamus	Sensory-motor cortex related
LGd	287458895	Thalamus	Sensory-motor cortex related
LGd	293787288	Thalamus	Sensory-motor cortex related
LGd	301735795	Thalamus	Sensory-motor cortex related
LGd	642480973	Thalamus	Sensory-motor cortex related
LGd	642180077	Thalamus	Sensory-motor cortex related
LGd	287458189	Thalamus	Sensory-motor cortex related
LGd	514513838	Thalamus	Sensory-motor cortex related

Experiments revised

Nuclei	Experiment nº	Structure	Region/Part/Area
LP	146658879	Thalamus	Sensory-motor cortex related
LP	572388249	Thalamus	Sensory-motor cortex related
LP	167439900	Thalamus	Sensory-motor cortex related
LP	504100025	Thalamus	Polymodal association cortex related
LP	507708083	Thalamus	Polymodal association cortex related
LP	292319865	Thalamus	Polymodal association cortex related
LP	166267651	Thalamus	Polymodal association cortex related
LP	183282970	Thalamus	Polymodal association cortex related
LP	266585624	Thalamus	Polymodal association cortex related
LP	183175010	Thalamus	Polymodal association cortex related
LP	267493760	Thalamus	Polymodal association cortex related
LP	183282261	Thalamus	Polymodal association cortex related
LP	293914056	Thalamus	Polymodal association cortex related
LP	301466249	Thalamus	Polymodal association cortex related
LP	183174303	Thalamus	Polymodal association cortex related
PO	267999740	Thalamus	Polymodal association cortex related
PO	183011353	Thalamus	Polymodal association cortex related
PO	174781014	Thalamus	Polymodal association cortex related
PO	100147785	Thalamus	Polymodal association cortex related
PO	292212456	Thalamus	Polymodal association cortex related
PO	182515576	Thalamus	Polymodal association cortex related
PO	182892855	Thalamus	Polymodal association cortex related
PO	180708524	Thalamus	Polymodal association cortex related
PO	301674988	Thalamus	Polymodal association cortex related
SGN	182805258	Thalamus	Polymodal association cortex related
SGN	113165340	Thalamus	Polymodal association cortex related
SGN	272873704	Thalamus	Polymodal association cortex related
SGN	300688721	Thalamus	Polymodal association cortex related
AV	267609756	Thalamus	Polymodal association cortex related
AV	100142569	Thalamus	Polymodal association cortex related
AV	479267539	Thalamus	Polymodal association cortex related
AV	175818392	Thalamus	Polymodal association cortex related
AV	292321278	Thalamus	Polymodal association cortex related
AV	114427219	Thalamus	Polymodal association cortex related
AV	146046430	Thalamus	Polymodal association cortex related
AV	286553311	Thalamus	Polymodal association cortex related
AV	292478008	Thalamus	Polymodal association cortex related
AV	614435699	Thalamus	Polymodal association cortex related
AV	182805965	Thalamus	Polymodal association cortex related
AV	605092364	Thalamus	Polymodal association cortex related
AM	158840459	Thalamus	Polymodal association cortex related
AM	167571459	Thalamus	Polymodal association cortex related
AM	146658170	Thalamus	Polymodal association cortex related
AM	506947040	Thalamus	Polymodal association cortex related
AM	514333422	Thalamus	Polymodal association cortex related
AM	266174045	Thalamus	Polymodal association cortex related
AM	573035760	Thalamus	Polymodal association cortex related

Experiments revised

Nuclei	Experiment nº	Structure	Region/Part/Area
AM	606278526	Thalamus	Polymodal association cortex related
AM	592698832	Thalamus	Polymodal association cortex related
AM	592698087	Thalamus	Polymodal association cortex related
AM	601900484	Thalamus	Polymodal association cortex related
AM	156393801	Thalamus	Polymodal association cortex related
IAD	168095041	Thalamus	Polymodal association cortex related
LD	267608343	Thalamus	Polymodal association cortex related
LD	305425490	Thalamus	Polymodal association cortex related
LD	175817683	Thalamus	Polymodal association cortex related
LD	113784293	Thalamus	Polymodal association cortex related
LD	272969333	Thalamus	Polymodal association cortex related
LD	113554008	Thalamus	Polymodal association cortex related
LD	272967913	Thalamus	Polymodal association cortex related
LD	309702727	Thalamus	Polymodal association cortex related
LD	298833033	Thalamus	Polymodal association cortex related
IMD	182185289	Thalamus	Polymodal association cortex related
IMD	299624500	Thalamus	Polymodal association cortex related
IMD	179902786	Thalamus	Polymodal association cortex related
MD	181895006	Thalamus	Polymodal association cortex related
MD	268076421	Thalamus	Polymodal association cortex related
MD	480703321	Thalamus	Polymodal association cortex related
MD	175739791	Thalamus	Polymodal association cortex related
MD	277849256	Thalamus	Polymodal association cortex related
MD	278067445	Thalamus	Polymodal association cortex related
MD	166264185	Thalamus	Polymodal association cortex related
MD	267928844	Thalamus	Polymodal association cortex related
MD	484504171	Thalamus	Polymodal association cortex related
MD	267610466	Thalamus	Polymodal association cortex related
MD	114291646	Thalamus	Polymodal association cortex related
MD	175740500	Thalamus	Polymodal association cortex related
MD	294356922	Thalamus	Polymodal association cortex related
MD	267607635	Thalamus	Polymodal association cortex related
MD	168300739	Thalamus	Polymodal association cortex related
MD	173206592	Thalamus	Polymodal association cortex related
MD	293367448	Thalamus	Polymodal association cortex related
MD	553747363	Thalamus	Polymodal association cortex related
MD	272875838	Thalamus	Polymodal association cortex related
MD	284665639	Thalamus	Polymodal association cortex related
MD	272830456	Thalamus	Polymodal association cortex related
MD	168301446	Thalamus	Polymodal association cortex related
MD	558697990	Thalamus	Polymodal association cortex related
MD	272875132	Thalamus	Polymodal association cortex related
MD	156931568	Thalamus	Polymodal association cortex related
MD	272970747	Thalamus	Polymodal association cortex related
MD	264707643	Thalamus	Polymodal association cortex related
MD	578332611	Thalamus	Polymodal association cortex related
MD	116904684	Thalamus	Polymodal association cortex related

Experiments revised

Nuclei	Experiment nº	Structure	Region/Part/Area
MD	524266253	Thalamus	Polymodal association cortex related
MD	268399145	Thalamus	Polymodal association cortex related
MD	168002780	Thalamus	Polymodal association cortex related
MD	278400363	Thalamus	Polymodal association cortex related
MD	168004394	Thalamus	Polymodal association cortex related
MD	183058837	Thalamus	Polymodal association cortex related
MD	183329222	Thalamus	Polymodal association cortex related
MD	286485585	Thalamus	Polymodal association cortex related
MD	301324895	Thalamus	Polymodal association cortex related
MD	267494468	Thalamus	Polymodal association cortex related
MD	300889379	Thalamus	Polymodal association cortex related
MD	301060890	Thalamus	Polymodal association cortex related
MD	609475139	Thalamus	Polymodal association cortex related
MD	293365328	Thalamus	Polymodal association cortex related
MD	286646170	Thalamus	Polymodal association cortex related
MD	298001595	Thalamus	Polymodal association cortex related
SMT	268163228	Thalamus	Polymodal association cortex related
SMT	147787606	Thalamus	Polymodal association cortex related
SMT	273025166	Thalamus	Polymodal association cortex related
PR	496965687	Thalamus	Polymodal association cortex related
PVT	278510903	Thalamus	Polymodal association cortex related
PVT	183225830	Thalamus	Polymodal association cortex related
PVT	313324664	Thalamus	Polymodal association cortex related
PVT	298050269	Thalamus	Polymodal association cortex related
PVT	127255962	Thalamus	Polymodal association cortex related
PVT	301209502	Thalamus	Polymodal association cortex related
PVT	184158996	Thalamus	Polymodal association cortex related
PVT	263106751	Thalamus	Polymodal association cortex related
PVT	120875111	Thalamus	Polymodal association cortex related
PVT	272874417	Thalamus	Polymodal association cortex related
PVT	184157585	Thalamus	Polymodal association cortex related
PVT	204908781	Thalamus	Polymodal association cortex related
PVT	299448592	Thalamus	Polymodal association cortex related
PT	159432479	Thalamus	Polymodal association cortex related
PT	159331462	Thalamus	Polymodal association cortex related
PT	123662982	Thalamus	Polymodal association cortex related
PT	305449231	Thalamus	Polymodal association cortex related
PT	176897793	Thalamus	Polymodal association cortex related
PT	305125123	Thalamus	Polymodal association cortex related
PT	167373923	Thalamus	Polymodal association cortex related
PT	266839077	Thalamus	Polymodal association cortex related
PT	545428296	Thalamus	Polymodal association cortex related
PT	171067319	Thalamus	Polymodal association cortex related
PT	180568155	Thalamus	Polymodal association cortex related
PT	126843200	Thalamus	Polymodal association cortex related
RE	174957972	Thalamus	Polymodal association cortex related
RE	175019536	Thalamus	Polymodal association cortex related

Experiments revised

Nuclei	Experiment nº	Structure	Region/Part/Area
RE	175374982	Thalamus	Polymodal association cortex related
RE	204832205	Thalamus	Polymodal association cortex related
RE	538833505	Thalamus	Polymodal association cortex related
RE	265286700	Thalamus	Polymodal association cortex related
RE	278500868	Thalamus	Polymodal association cortex related
RE	310976160	Thalamus	Polymodal association cortex related
RE	184158290	Thalamus	Polymodal association cortex related
RE	265947058	Thalamus	Polymodal association cortex related
RE	607316031	Thalamus	Polymodal association cortex related
RE	170784358	Thalamus	Polymodal association cortex related
RE	496964969	Thalamus	Polymodal association cortex related
RE	607289053	Thalamus	Polymodal association cortex related
RE	528732005	Thalamus	Polymodal association cortex related
RE	294040662	Thalamus	Polymodal association cortex related
RE	113504763	Thalamus	Polymodal association cortex related
RE	167025578	Thalamus	Polymodal association cortex related
CM	301538025	Thalamus	Polymodal association cortex related
CM	158841171	Thalamus	Polymodal association cortex related
CM	303710632	Thalamus	Polymodal association cortex related
CM	147135107	Thalamus	Polymodal association cortex related
CM	183057424	Thalamus	Polymodal association cortex related
CM	182888003	Thalamus	Polymodal association cortex related
PCN	183071513	Thalamus	Polymodal association cortex related
PF	267959197	Thalamus	Polymodal association cortex related
PF	263785543	Thalamus	Polymodal association cortex related
PF	299732738	Thalamus	Polymodal association cortex related
PF	300642574	Thalamus	Polymodal association cortex related
PF	272968624	Thalamus	Polymodal association cortex related
PF	266693274	Thalamus	Polymodal association cortex related
PF	309580808	Thalamus	Polymodal association cortex related
PIL	127396051	Thalamus	Polymodal association cortex related
PIL	586054741	Thalamus	Polymodal association cortex related
PIL	287880821	Thalamus	Polymodal association cortex related
PIL	301063301	Thalamus	Polymodal association cortex related
RT	127090378	Thalamus	Polymodal association cortex related
RT	117316260	Thalamus	Polymodal association cortex related
RT	299894738	Thalamus	Polymodal association cortex related
RT	127468854	Thalamus	Polymodal association cortex related
RT	171065200	Thalamus	Polymodal association cortex related
RT	287446625	Thalamus	Polymodal association cortex related
RT	552973699	Thalamus	Polymodal association cortex related
RT	127085005	Thalamus	Polymodal association cortex related
RT	292960052	Thalamus	Polymodal association cortex related
RT	156252954	Thalamus	Polymodal association cortex related
RT	171064488	Thalamus	Polymodal association cortex related
RT	301421253	Thalamus	Polymodal association cortex related
RT	554651619	Thalamus	Polymodal association cortex related

Experiments revised

Nuclei	Experiment nº	Structure	Region/Part/Area
RT	156254369	Thalamus	Polymodal association cortex related
LGv	267538006	Thalamus	Polymodal association cortex related
LGv	147212977	Thalamus	Polymodal association cortex related
LGv	299623794	Thalamus	Polymodal association cortex related
LGv	525796603	Thalamus	Polymodal association cortex related
LGv	157765542	Thalamus	Polymodal association cortex related
LGv	278398949	Thalamus	Polymodal association cortex related
LGv	301991713	Thalamus	Polymodal association cortex related
MH	300236056	Thalamus	Polymodal association cortex related
MH	300843826	Thalamus	Polymodal association cortex related
MH	268321927	Thalamus	Polymodal association cortex related
MH	265287564	Thalamus	Polymodal association cortex related
LH	292623457	Thalamus	Polymodal association cortex related
LH	147353537	Thalamus	Polymodal association cortex related
LH	301057735	Thalamus	Polymodal association cortex related
LH	551351756	Thalamus	Polymodal association cortex related
LH	510125001	Thalamus	Polymodal association cortex related
LH	510124187	Thalamus	Polymodal association cortex related
	**Total**	**Thalamus**	**268**

Experiments reviewed	
Pallidum	**67**
Septal	**30**
Striatum	**131**
Hypothalamus	**258**
Thalamus	**268**
**Total**	**754**
